# NUSAP1 promotes invasion and metastasis of prostate cancer

**DOI:** 10.18632/oncotarget.15604

**Published:** 2017-02-22

**Authors:** Catherine A. Gordon, Xue Gong, Durga Ganesh, James D. Brooks

**Affiliations:** ^1^ Department of Urology, Stanford University School of Medicine, Stanford, CA, 94305, USA; ^2^ Department of Pathology, Stanford University School of Medicine, Stanford, CA, 94305, USA; ^3^ Stanford Cancer Institute, Stanford University School of Medicine, Stanford, CA, 94305, USA

**Keywords:** NUSAP1, invasion, migration, metastasis, prostate cancer

## Abstract

We have previously identified nucleolar and spindle associated protein 1 (*NUSAP1*) as a prognostic biomarker in early stage prostate cancer. To better understand the role of NUSAP1 in prostate cancer progression, we tested the effects of increased and decreased *NUSAP1* expression in cell lines, *in vivo* models, and patient samples. NUSAP1 promotes invasion, migration, and metastasis, possibly by modulating family with sequence similarity 101 member B (*FAM101B*), a transforming growth factor beta 1 (TGFβ1) signaling effector involved in the epithelial to mesenchymal transition. Our findings provide insights into the importance of NUSAP1 in prostate cancer progression and provide a rationale for further study of NUSAP1 function, regulation, and clinical utility.

## INTRODUCTION

Considerable controversy and confusion surround the diagnosis and management of clinically localized prostate cancer, the most common malignancy in men in the USA and Europe [1]. Widespread screening for prostate cancer in the USA has been associated with a 75% drop in metastatic disease at presentation [2], and randomized trials in Europe show survival benefits to PSA screening and prostate surgery [3, 4]. However, randomized trials of screening and surgery in the USA have shown no survival benefit [5, 6], and a consensus has developed that localized prostate cancer is over-diagnosed and over-treated, even in the European randomized trials. In response to these conflicting data, some recommend against screening and treatment of clinically localized prostate cancer [7], others advocate for increased use of active surveillance [8–10], while others seek to develop approaches to better predict the natural history of localized prostate cancer to better select men who need aggressive therapies.

Previously, we identified *NUSAP1* as a candidate prognostic biomarker in patients undergoing radical prostatectomy [11]. In several diverse datasets, increased *NUSAP1* expression is associated with recurrence after radical prostatectomy [11], and it is found in prognostic gene sets associated with high grade compared to low grade prostate cancer [12], two commercial platforms used to predict prognosis (Prolaris from Myriad Genetics and Decipher from GenomeDx Biosciences) [13–16], and a set of transcripts upregulated in a model of castration resistant prostate cancer [17]. *NUSAP1* overexpression is also prognostic in other cancer types, including melanoma [18, 19], breast cancer [20, 21], glioblastoma [22], hepatocellular carcinoma [23], and meningioma [24]. The breadth of studies implicating NUSAP1 suggests it plays an important functional role in cancer progression.

Current understanding of NUSAP1's function is limited, although existing data suggests it could simply be a marker of proliferation [25]. NUSAP1 is an essential microtubule and chromatin-binding protein that cross-links microtubules during mitosis [26–28], modulates the dynamics of kinetochore microtubules [29], and governs chromosome oscillation [30]. *NUSAP1* expression is regulated by E2F transcription factor 1 (E2F1) and by loss of retinoblastoma-associated protein 1 (RB1), an important molecular pathway that becomes altered in aggressive forms of prostate cancer [11, 31, 32]. To better understand the functional role of NUSAP1 in prostate cancer, we explored the effects of overexpression and knockdown of *NUSAP1* in *in vitro* and *in vivo* models. We find that NUSAP1 has limited effects on proliferation, but rather is associated with development of metastatic disease, possibly through modulation of expression of *FAM101B*. FAM101B is involved in cell shape remodeling during the epithelial to mesenchymal transition and is a signaling effector of TGFβ1 [33, 34], which promotes invasion and metastatic spread during prostate tumor progression [35].

## RESULTS

### NUSAP1 promotes invasion and migration

**Figure 1:**
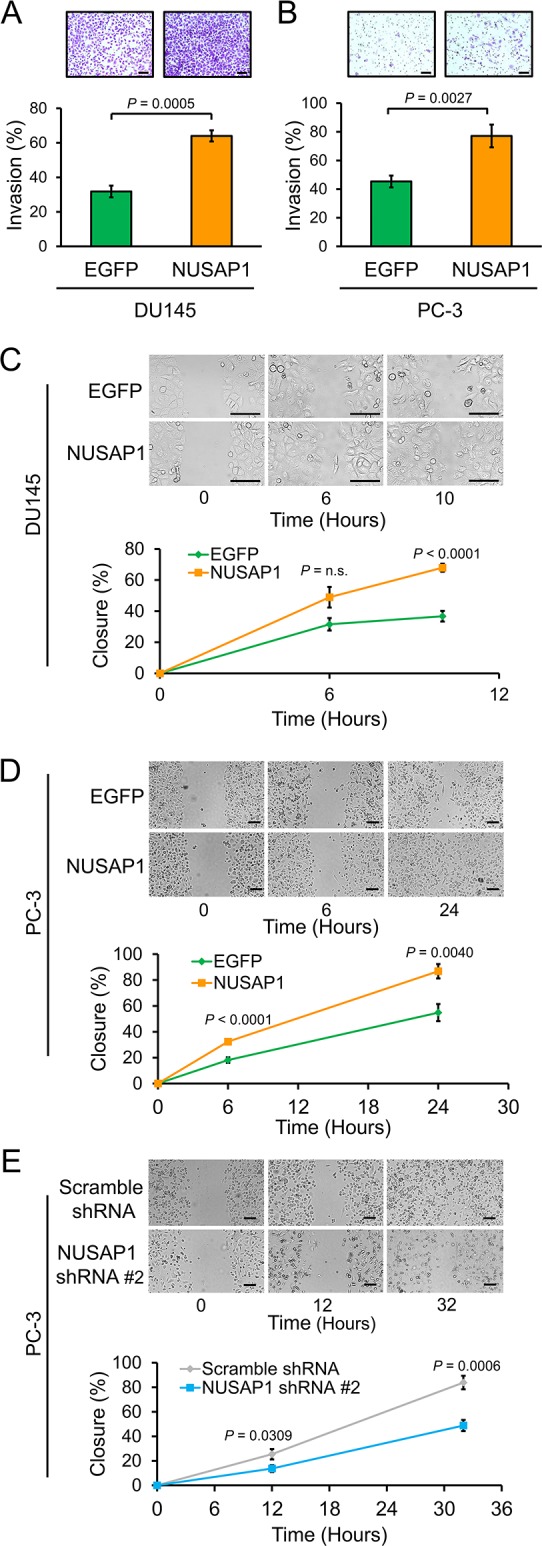
*NUSAP1* overexpression increases invasion and migration of prostate cancer cells **A** and **B**. Matrigel chambers were used to perform invasion assays with (A) DU145 and (B) PC-3 cells stably overexpressing *NUSAP1* or *EGFP* control. Representative images show invasion of Matrigel membranes stained with 0.5% crystal violet. Bars: mean ± SEM (standard error of the mean). **C**, **D**, and **E**. Confluent monolayers of (C) DU145 and (D and E) PC-3 cells stably overexpressing or underexpressing *NUSAP1* or controls were abraded and then monitored over time for wound channel closure. Representative images show wound channel closure over time. The migratory rate was determined by measuring wound channel area as a function of time using NIH Image J software. Points: mean ± SEM; n.s.: not significant. All scale bars: 100 μm. All *P*-values were calculated using the two-tailed Student's t-test.

We previously found that *NUSAP1* is overexpressed in recurrent prostate cancer and is regulated, at least in part, by loss of RB1 via the RB1/E2F1 axis [11, 31]. Since knockdown of *NUSAP1* results in reduced invasion of PC-3 prostate cancer cells [11], we hypothesized that overexpression of *NUSAP1* might have the opposite effect, leading to increased invasion of prostate cancer cells. To test this hypothesis, we used lentiviral infections to overexpress *NUSAP1* or *EGFP* control in DU145 or PC-3 cells, verified overexpression by reverse transcription-quantitative polymerase chain reaction (RT-qPCR) and western blot (Supplementary Figures 1A and 1B), and plated cells in Matrigel invasion chambers (BD Biosciences, Franklin Lakes, NJ, USA). Overexpression of *NUSAP1* increased invasion of both DU145 and PC-3 cells compared to controls (Figure 1A and 1B). Furthermore, wound healing assays showed that overexpression of *NUSAP1* (Supplementary Figures 1A and 1B) led to increased migration, while knockdown of *NUSAP1* (Supplementary Figure 1B) led to reduced migration of DU145 or PC-3 cells (Figures 1C-1E).

NUSAP1 binds DNA to the mitotic spindle and we have shown that knockdown of *NUSAP1* results in reduced proliferation of DU145, LNCaP, PC-3, and PC-3-RB1 low prostate cancer cells grown in culture [11, 31], yet it was unknown whether *NUSAP1* overexpression would enhance proliferation. Overexpression of *NUSAP1* or *EGFP* control in DU145, LNCaP, PC-3, or 22Rv1 prostate cancer cell lines using a lentiviral vector (Supplementary Figures 1A-1D) did not significantly affect proliferation rates over a period of 5 days (Supplementary Figures 2A-2D). Flow cytometry confirmed that overexpression of *NUSAP1* did not significantly alter cell cycle stages or apoptosis (Supplementary Figures 3A and 3B); however, knockdown of *NUSAP1* (Supplementary Figures 1B and 1C) resulted in a significant increase in the number of cells in the G2/M phase of the cell cycle and number of apoptotic cells (Supplementary Figures 3C and 3D).

**Figure 2:**
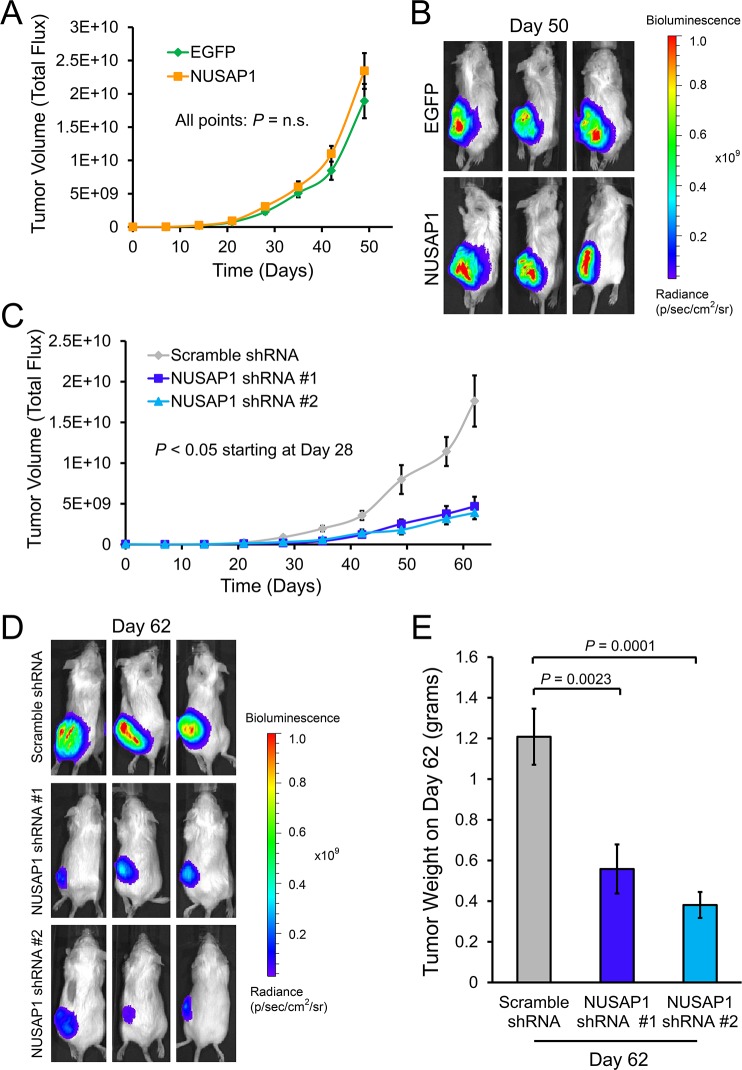
Effects of *NUSAP1* overexpression or underexpression on tumor volume as measured by bioluminescence **A**. Bioluminescence measurements of tumor volume over time in mice with tumors overexpressing *NUSAP1* versus *EGFP*. Points: mean ± SEM. EGFP: n = 23. NUSAP1: n = 23. **B**. Representative images of tumor bioluminescence on day 50 in mice with tumors overexpressing *NUSAP1* versus *EGFP* control. **C**. Bioluminescence measurements of tumor volume over time in mice with tumors underexpressing *NUSAP1* versus control. Points: mean ± SEM. Scramble shRNA: n = 12; NUSAP1 shRNA #1: n = 10; NUSAP1 shRNA #2: n = 9. **D**. Representative images of tumor bioluminescence on day 62 in mice with tumors expressing scramble shRNA, *NUSAP1* shRNA #1, or *NUSAP1* shRNA #2. **E**. Average tumor weights of mice on day 62 with tumors expressing scramble shRNA, *NUSAP1* shRNA #1, or *NUSAP1* shRNA #2. Bars: mean ± SEM. Scramble shRNA: n = 12; NUSAP1 shRNA #1: n = 11; NUSAP1 shRNA #2: n = 9. All *P*-values were calculated using the two-tailed Student's t-test.

To further determine if *NUSAP1* expression would affect proliferation *in vivo*, we used lentiviral infections to overexpress or knockdown *NUSAP1* or controls in PC-3-luc2 cells (PC-3 cells that stably express the firefly luciferase gene), verified overexpression or knockdown of *NUSAP1* by RT-qPCR and western blot (Supplementary Figure 1E), and tested whether *NUSAP1* overexpression or knockdown affected growth of PC-3-luc2 cells in mouse xenografts. When measuring tumor volume using total flux bioluminescence, there was no significant difference in bioluminescence of *NUSAP1* overexpressing versus *EGFP* overexpressing subcutaneous tumors by 50 days (Figure 2A and 2B). Caliper measurements, however, demonstrated a relatively small but significant difference in the size of *NUSAP1* overexpressing versus *EGFP* overexpressing subcutaneous tumors by 41 days (Supplementary Figure 4A). After harvesting tumors, RT-qPCR demonstrated continued overexpression in the lentiviral transfected tumors (Supplementary Figure 4B). Knockdown of *NUSAP1* expression produced a significant reduction in tumor size measured by bioluminescence and calipers (Figures 2C, 2D, and Supplementary Figure 4C). After harvesting tumors, RT-qPCR demonstrated continued knockdown in the lentiviral transfected tumors when using NUSAP1 shRNA #2, but no significant difference when using NUSAP1 shRNA #1 (Supplementary Figure 4D). Tumor weights were also lower when *NUSAP1* had been knocked down (Figure 2E), consistent with decreased tumor volume and reduced proliferation.

### NUSAP1 is associated with metastasis in patient samples and promotes metastasis in mice

**Figure 3:**
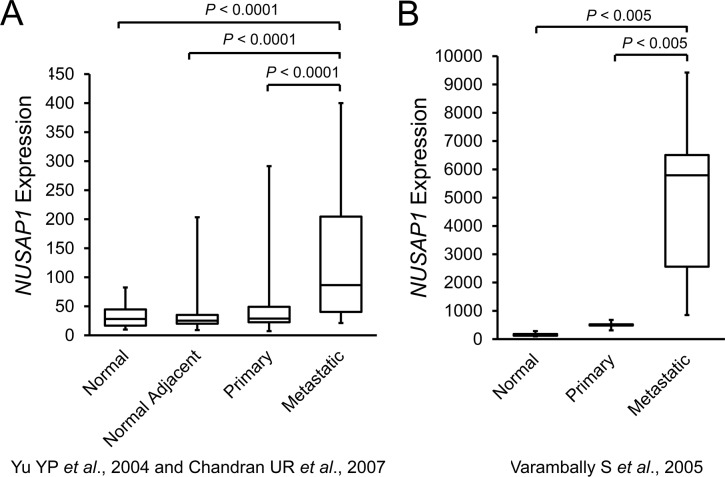
*NUSAP1* transcripts are increased in patient metastatic prostate tumors **A**. Box plots of *NUSAP1* expression in the Yu YP *et al*., 2004 [36] and Chandran UR *et al*., 2007 [37] datasets (GEO Accession: GDS2547; GEO Profile: 34888857). Normal: n = 17; Normal Adjacent: n = 58; Primary: n = 64; Metastatic: n = 25. **B**. Box plots of *NUSAP1* expression in the Varambally S *et al*., 2005 [38] dataset (GEO Accession: GDS1439; GEO Profile: 14252725). Normal: n = 6; Primary: n = 7; Metastatic: n = 6. All *P*-values were calculated using the two-tailed Student's t-test.

Since overexpression of *NUSAP1* is sufficient to result in increased invasion and migration, we used publicly available gene expression datasets (www.ncbi.nlm.nih.gov/geo/) to investigate the relative levels of gene expression in normal prostate tissue, primary prostate cancer, and metastatic prostate cancer [36–38]. Intriguingly, *NUSAP1* transcripts were highest in metastatic samples and lowest in noncancerous samples (Figure 3A and 3B). Based on this observation, we tested whether *NUSAP1* overexpression would lead to increased metastasis while *NUSAP1* knockdown would lead to reduced metastasis in our mouse xenograft models. By *ex vivo* bioluminescence imaging, we measured luciferase expression in each lung, right axillary lymph node, and left axillary lymph node of sacrificed mice that had *NUSAP1* overexpression or knockdown in flank tumors. In this relatively small set of mouse xenografts, overexpression of *NUSAP1* was associated with a relative increase in the percentage of mice with metastases, while knockdown of *NUSAP1* was associated with a reduction (Supplementary Table 1).

**Figure 4:**
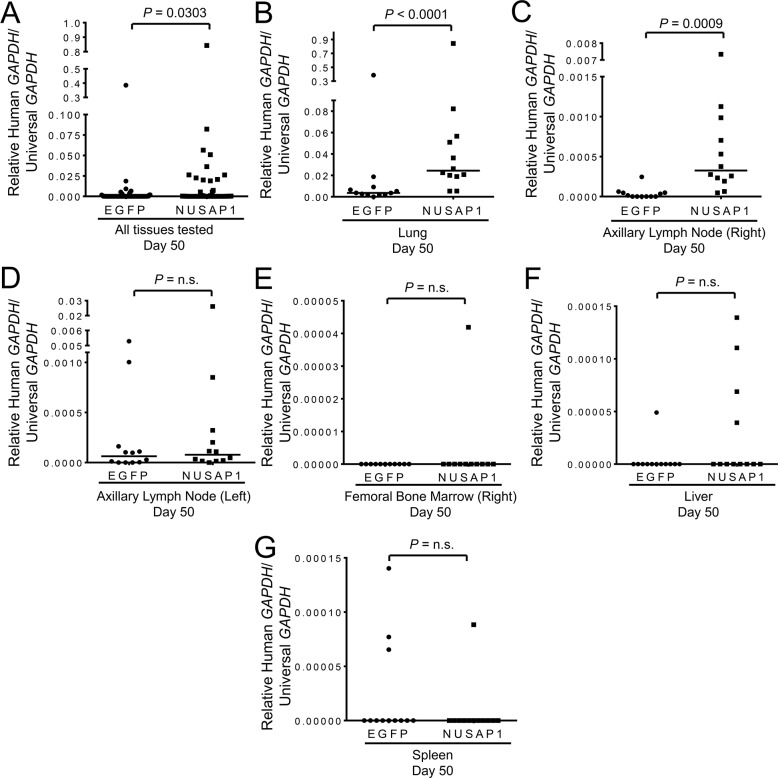
*NUSAP1* overexpression significantly increases metastases in a PC-3-luc2 xenograft model Mice bearing PC-3-luc2 flank tumors overexpressing *NUSAP1* or *EGFP* were assessed for metastases to the lung, axillary lymph nodes (right and left), femoral bone marrow (right), liver, or spleen by measuring expression levels of human *GAPDH* relative to universal *GAPDH* by RT-qPCR. Measurements of metastases in **A**. all six tissue sites combined and the **B**. lung, **C**. right axillary lymph node, **D**. left axillary lymph node, **E**. right femoral bone marrow, **F**. liver, and **G**. spleen. All horizontal lines represent the median. All *P*-values were calculated using the Mann-Whitney U test.

We then generated additional mouse xenografts of PC-3-luc2 cells in which *NUSAP1* or controls were overexpressed or knocked down. To quantitatively assess the extent of metastases, we used RT-qPCR for human-specific *GAPDH* expression versus universal (both human and mouse) *GAPDH* expression as described previously [39], and tested relative *GAPDH* transcript levels in each lung, right axillary lymph node, left axillary lymph node, right femoral bone marrow, liver, and spleen. For all tissue sites combined, the percentage of mice with metastases was significantly higher when *NUSAP1* was overexpressed in the primary tumor compared to *EGFP* overexpression (Supplementary Figure 5A). When looking at individual tissues, the percentage of mice with metastases was also higher when *NUSAP1* was overexpressed in each tissue examined except for the spleen (Supplementary Figures 5B-5G). Likewise, the human *GAPDH*/universal *GAPDH* transcript levels, a surrogate for metastatic burden, were higher, overall, in *NUSAP1* overexpressing tumors (Figure 4A). Interestingly, *NUSAP1* overexpression resulted in a statistically significant increase in the quantity of metastases found in the lungs and right axillary lymph nodes (Figure 4B and 4C), but not in other tissues examined (Figures 4D-4G).

### *NUSAP1* overexpression or knockdown leads to differentially expressed genes involved in organismal injury and abnormalities, cancer, and cell death and survival

**Figure 5:**
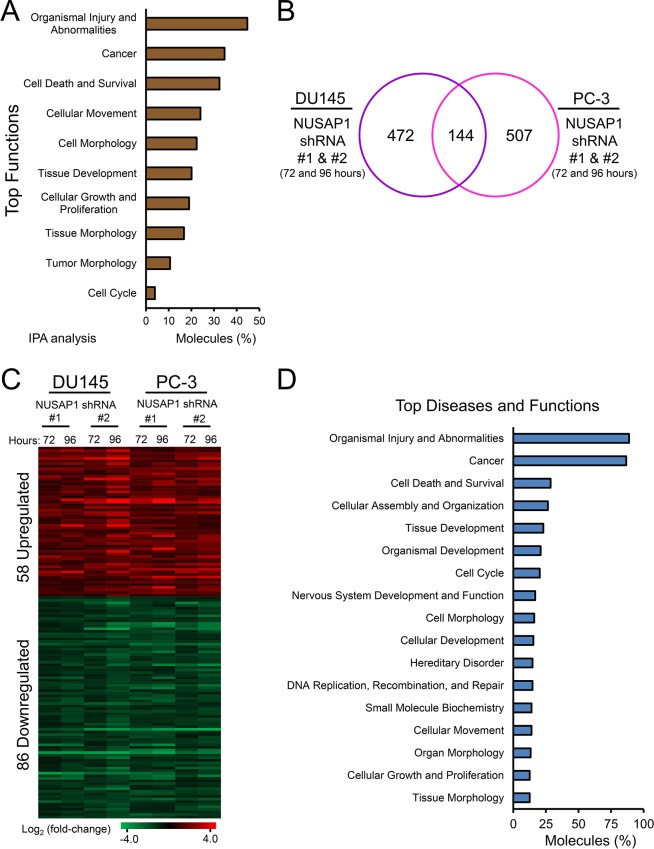
*NUSAP1* overexpression or knockdown leads to differentially expressed genes associated with cancer progression **A**. Graph illustrates enriched functions of the 185 differentially expressed genes when *NUSAP1* is overexpressed in PC-3 cells as determined by Ingenuity Pathway Analysis. **B**. A common 144-member gene signature of differentially expressed transcripts is revealed after *NUSAP1* knockdown in DU145 and PC-3 cells (fold-change ≥ 1.5 and adjusted *P* < 0.05). **C**. Heatmap of the 144-member gene signature. **D**. Graph illustrates enriched functions of the 144 differentially expressed genes when *NUSAP1* is knocked down in DU145 and PC-3 cells as determined by Ingenuity Pathways Analysis.

To gain insights into gene expression alterations induced when NUSAP1 is modulated, we used lentiviral infections to overexpress or knockdown *NUSAP1* in DU145 or PC-3 cells for 72 or 96 hours, verified overexpression or knockdown by RT-qPCR and western blot (Supplementary Figures 1A and 1B), and performed RNA sequencing (RNA-Seq). When *NUSAP1* was overexpressed in PC-3 cells for 96 hours, RNA-Seq showed that *NUSAP1* was overexpressed 3.1-fold and was associated with 185 differentially expressed genes (78 upregulated and 107 downregulated) compared to *EGFP* control cells (fold-change ≥ 1.5 and adjusted *P* < 0.05; Supplementary Table 2). Ingenuity Pathway Analysis revealed that tumor progression was predicted to increase based on the direction of differentially expressed genes (*P* = 0.0002; Z-score = 2.390). In addition, Ingenuity Pathway Analysis revealed that the differentially expressed genes were involved in functions that include cancer, cellular movement, and cell morphology (Figure 5A).

Knockdown of *NUSAP1* expression in DU145 or PC-3 cells stably using two different shRNAs to *NUSAP1* versus scramble shRNA control was performed for 72 or 96 hours post initial infection. RNA-Seq showed that *NUSAP1* knockdown decreased *NUSAP1* expression 3.1-fold to 22.6-fold compared to control, and revealed 144 transcripts (58 upregulated and 86 downregulated) that were modulated in all eight knockdown experiments (Figures 5B and 5C and Supplementary Table 3). This 144-member gene signature was associated with functions that include cancer, cellular assembly and organization, and tissue development (Figure 5D). Interestingly, the top three functions (organismal injury and abnormalities, cancer, and cell death and survival) were the same in the overexpression and knockdown experiments (Figure 5A and 5D).

**Figure 6:**
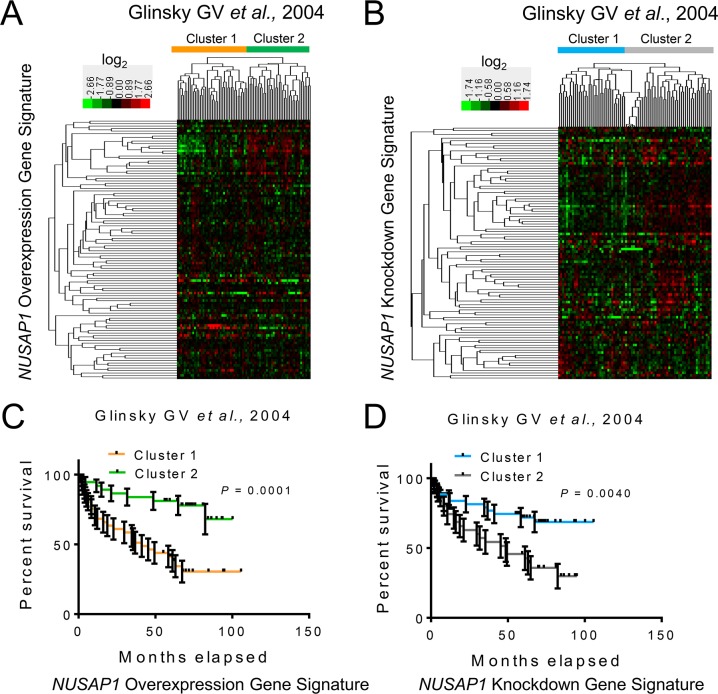
*NUSAP1* overexpression and knockdown gene signatures are prognostic in human prostate cancer samples **A** and **B**. Hierarchical clustering of the Glinsky GV *et al*., 2004 [41] prostate cancer samples across the (A) 104 genes affected by *NUSAP1* overexpression and the (B) 100 genes affected by *NUSAP1* knockdown. (A) Orange and green bars represent the two clusters when using the *NUSAP1* overexpression gene signature. (B) Blue and gray bars represent the two clusters when using the *NUSAP1* knockdown gene signature. **C** and **D**. Kaplan-Meier survival analysis comparing the two clusters from the *NUSAP1* (C) overexpression and (D) knockdown gene signatures. Error bars: ± SEM. *P*-values were calculated using the log-rank test.

To determine whether the gene signatures modulated by *NUSAP1* overexpression or knockdown in prostate cancer cell lines relate to the behavior of human prostate cancers, we used the Significance Analysis of Microarrays technique (SAM) [40] to identify genes positively and negatively correlated with *NUSAP1* in a prostate cancer dataset with well annotated clinical follow-up [41]. We identified 3,414 transcripts correlated with *NUSAP1* expression (2,610 positively correlated and 804 negatively correlated; FDR = 0.0455). Of the 185 transcripts found in *NUSAP1* overexpressing PC-3 cells in culture, 104 were among the 3,414 transcripts and were both significantly enriched and differentially expressed in the same direction (Hypergeometric test; *P* = 0.0152). Of the 144 transcripts differentially expressed upon *NUSAP1* knockdown in cell culture, 100 of them were found among the 3,414 transcripts and were both significantly enriched and differentially expressed in the same direction (Hypergeometric test; *P* = 0.0013). Furthermore, when we used the 104 and 100 gene sets to perform unsupervised hierarchical clustering, patient samples segregated into two groups (Figure 6A and 6B) that displayed significantly different patient survival (Figure 6C and 6D). Notably, the worse outcome group from the *NUSAP1* overexpression gene signature (cluster 1 in Figure 6C) showed significant enrichment for expression in the same direction (increased or decreased expression) as when *NUSAP1* was overexpressed *in vitro*, while the better outcome group (cluster 2 in Figure 6C) was inversely correlated with the direction of gene expression (Fisher's exact test; *P* < 0.001). Likewise, the better outcome group from the *NUSAP1* knockdown signature (cluster 1 in Figure 6D) showed significant enrichment for expression in the same direction as the *in vitro* experiments, while the worse outcome group (cluster 2 in Figure 6D) was inversely correlated to the knockdown gene expression direction (Fisher's exact test; *P* < 0.001).

### FAM101B is a potential downstream effector of NUSAP1

**Figure 7:**
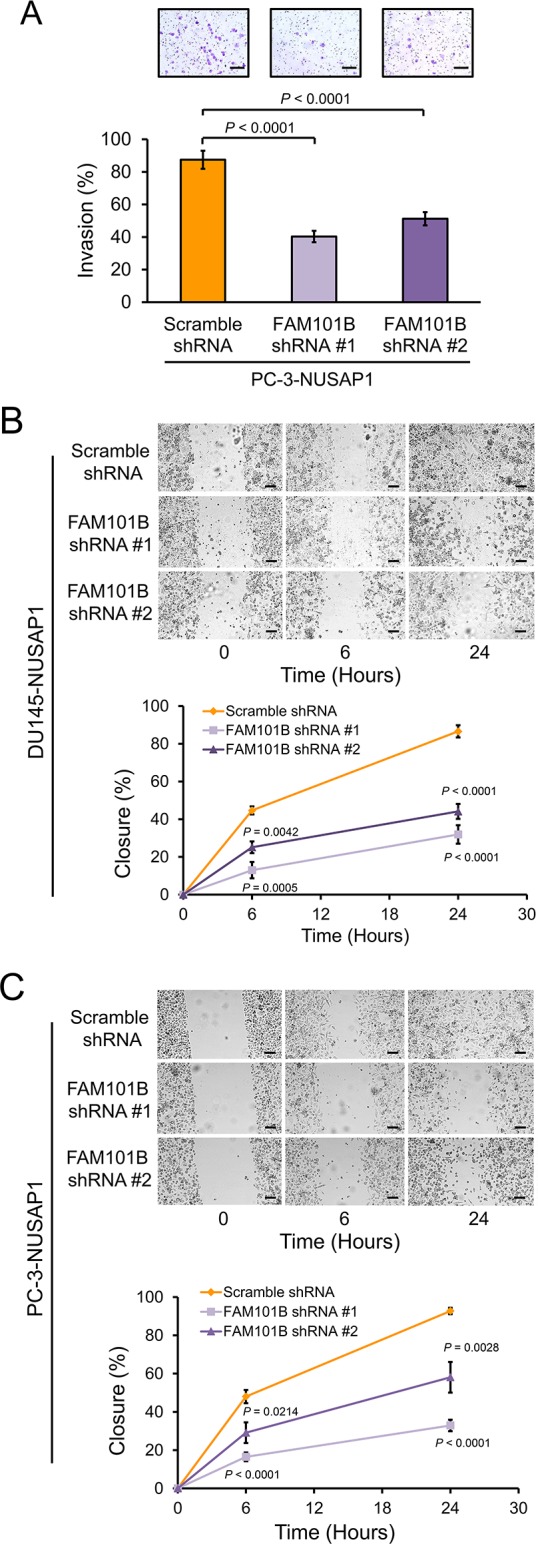
Knockdown of *FAM101B* decreases invasion and migration of prostate cancer cells **A**. Invasion assays using Matrigel invasion chambers after lentiviral shRNA knockdown of *FAM101B* in PC-3-NUSAP1 cells. Representative images show invasion of Matrigel membranes stained with 0.5% crystal violet. Bars: mean ± SEM. **B** and **C**. Wound healing assays in (B) DU145-NUSAP1 and (C) PC-3-NUSAP1 cells after knockdown of *FAM101B* versus control. Representative images show wound channel closure over time. The migratory rate was determined by measuring wound channel area as a function of time using NIH Image J software. Points: mean ± SEM. All scale bars: 100 μm. All *P*-values were calculated using the two-tailed Student's t-test.

Comparison of the *NUSAP1* overexpression and knockdown datasets showed that *FAM101B* was induced in response to *NUSAP1* overexpression and suppressed when *NUSAP1* was knocked down. FAM101B is a filamin binding protein that is upregulated by TGFβ1, participates in skeletal development, and is involved in cell shape remodeling during the epithelial to mesenchymal transition [33, 34]. Since FAM101B is involved in pathways involved in cancer cell invasion and metastasis, we wondered if FAM101B might play a role downstream of NUSAP1 in making prostate cancer cells more aggressive. After verifying that *FAM101B* expression is upregulated in DU145, PC-3, and PC-3-luc2 cells overexpressing *NUSAP1* (Supplementary Figures 6A-6C), we knocked down *FAM101B* expression in DU145-NUSAP1 (DU145 cells stably overexpressing *NUSAP1*) and PC-3-NUSAP1 (PC-3 cells stably overexpressing *NUSAP1*) cells using two different shRNAs (Supplementary Figures 6D and 6E). Invasion and wound healing assays showed that knockdown of *FAM101B* reversed the effects of *NUSAP1* overexpression, leading to reduced invasion (Figure 7A) and migration rates (Figure 7B and 7C). In patient gene expression datasets, *FAM101B* expression was significantly elevated in metastatic tumors of the Yu YP *et al*. and Chandran UR *et al*. datasets [36, 37] (Supplementary Figure 6F), but not in the metastatic tumor samples from Varambally S *et al*. [38], although the latter dataset included only 6 patient samples (Supplementary Figure 6G). Furthermore, in the gene expression dataset from Taylor *et al*. [42], where we have previously shown that *NUSAP1* expression levels are significantly correlated with recurrence after surgery [11], *FAM101B* and *NUSAP1* gene expression levels in all 216 tumors had a tendency to co-occur (Log Odds Ratio = 0.969; Z-score ± 2.0; www.cbioportal.org/) [43, 44].

## DISCUSSION

While NUSAP1 has been linked to proliferation based on its role in assembly of the mitotic spindle, our results provide compelling evidence that NUSAP1 plays a direct role in driving prostate cancer progression. In prostate cancer cell lines *in vitro*, overexpression of *NUSAP1* did not increase proliferation, but did increase invasion and migration, and depletion of *NUSAP1* decreased proliferation, invasion, and migration. Modest *NUSAP1* overexpression in prostate cancer xenografts, comparable to levels observed in human prostate cancers, significantly increased metastases and modestly affected tumor volume. Consistent with these findings, analysis of human prostate cancer samples showed that *NUSAP1* is highly expressed in metastases, compared to localized tumors. Overexpression and knockdown of *NUSAP1* produced changes in gene expression programs associated with tumor progression, and these gene sets predict patient outcomes in patients undergoing surgery for localized prostate cancer. One gene consistently modulated, *FAM101B*, correlates with *NUSAP1* expression in patient samples and appears to be an important downstream effector of NUSAP1 in tumor progression since knockdown of *FAM101B* abolished NUSAP1's effects on prostate cancer cell invasion and migration. Our data suggests a direct functional role of NUSAP1 in tumor progression, bolstering its role as a prognostic biomarker and warranting further investigation into its function and therapeutic potential.

Elevated *NUSAP1* expression is associated with increased aggressiveness and poor patient outcome in prostate cancer [11]. Loss of RB1 is common in prostate cancer [45–47], controls progression into castration-resistant prostate cancer [32, 45–47], and is a mechanism responsible for overexpression of *NUSAP1* [31]. Overexpression of androgen receptor (AR) is a common event in castration-resistant prostate cancer [48–52] and may be directly related to overexpression of *NUSAP1*. In LNCaP cells that stably overexpress *AR*, *NUSAP1* expression is significantly increased [53]. Although a putative AR binding site in the *NUSAP1* promoter has not been identified, an AR binding site approximately 100 kb upstream of the *NUSAP1* transcription start site has been reported [53], suggesting that AR might regulate *NUSAP1* expression via this enhancer region. *NUSAP1* expression increases in response to androgen treatment in LNCaP cells [54]; however, NUSAP1 is associated with the mitotic spindle and androgen treatment of prostate epithelial cells induces proliferation. Further testing is necessary to determine if there is a direct connection between AR and *NUSAP1* expression.

Surprisingly, little work has examined the role of NUSAP1 in cancer, despite several reports documenting that overexpression of *NUSAP1* is associated with significantly worse outcome [55]. Given its critical role in mitosis, knockdown of *NUSAP1* results in decreased proliferation, cell cycle arrest in G2/M, and induction of apoptosis [26, 56, 57], as we have observed here. Transient high-level overexpression of *NUSAP1* can also be toxic to cells [26, 56], limiting previous efforts to study its potential role in carcinogenesis and progression. Unlike previous reports, we used lentiviral infections, rather than transfections, to overexpress *NUSAP1*. This approach resulted in a relatively lower level of *NUSAP1* expression, demonstrated by RT-qPCR, western blot, and RNA-Seq, such that the levels of overexpression were in the same range as has been observed in human prostate cancers and other malignancies. Very likely, this lower level of induction was due to selective outgrowth of cells that had fewer copies of *NUSAP1* cDNA per cell. Regardless, the modest inductions we achieved allowed us to investigate the effects *NUSAP1* overexpression.

Although previous studies have suggested that knockdown of *NUSAP1* decreases invasion of cells *in vitro* [11, 31], the effects of knockdown on cell growth, including cell cycle arrest and apoptosis, could confound determination of a direct role of NUSAP1 in tumor progression since dying or arrested cells are less likely to invade. However, since modest increases in expression were achieved and tolerated in our prostate cancer cell lines, we were able to demonstrate that increased *NUSAP1* expression increases invasion and migration *in vitro*, and metastasis *in vivo*. These modest increases in *NUSAP1* levels had no effect on proliferation *in vitro* and equivocal results on tumor growth in mice. Only one previous study in zebrafish has linked *nusap1* expression to cell migration and is consistent with our findings. When expression of *nusap1* was depleted in zebrafish embryos by antisense oligonucleotide morpholino microinjection, extensions of the trunk and yolk were impaired, and this impairment was caused by significantly decreased neural crest cell migration [58]. While our effects on metastases are modest, they are consistent with the natural history of prostate cancer, which is slowly progressive and with low proliferative index. The finding of increased *NUSAP1* expression levels in metastatic prostate cancer argues strongly for its role in progression.

Modulation of *NUSAP1* expression produced changes in gene expression that correlate with the observed changes in invasion, migration, metastases, and clinical outcome. The gene expression patterns elicited by *NUSAP1* overexpression or knockdown fall into a remarkably consistent set of pathways related to tumor progression. Interestingly, we identified FAM101B, a TGFβ1 signaling effector [33], as a potential downstream effector of NUSAP1 in tumor progression. TGFβ1 is a master regulator of the epithelial to mesenchymal transition, a process that enables tumor invasion and cancer metastasis [59]. Depletion of *FAM101B* expression in *NUSAP1* overexpressing cell lines abolished the effect of increased invasion and migration that results when *NUSAP1* is overexpressed. Like *NUSAP1*, we found that *FAM101B* expression is significantly increased in metastatic patient samples compared to non-cancerous and localized cancer patient samples and correlates with *NUSAP1* expression in localized prostate cancer. *FAM101B* transcripts are also found at medium to high levels in several different cancer cell lines, including neuroblastoma, melanoma, cervical squamous carcinoma, endometrial adenocarcinoma, choriocarcinoma, epidermoid carcinoma, and lymphoma and leukemia cell lines (www.proteinatlas.org/) [60]. Furthermore, FAM101B protein levels are found at medium to high levels in human breast, renal, testis, and prostate cancers [60]. In general, *FAM101B* and *NUSAP1* expression correlate in the cell lines and cancers tested; however, it should be noted that medium to high levels of *NUSAP1* expression are found in additional and most cell lines and cancers tested [60]. Our analyses suggest *NUSAP1* overexpression may lead to tumor progression in patients, at least in part, via FAM101B. Additional work will be necessary to understand the mechanisms underlying NUSAP1's effects on gene expression and tumor progression. NUSAP1 contains a DNA binding domain [27, 61], so it is possible that NUSAP1 directly acts as a transcriptional regulator.

*NUSAP1* is a highly validated biomarker of prostate cancer progression [11, 13–16] and overexpressed in multiple cancer types [55]. Our analyses of prostate cancer cell lines and prostate cancer patient samples suggest that *NUSAP1* is more than just a prognostic biomarker in prostate cancer, but actually plays a role in driving prostate cancer progression. NUSAP1 appears to drive prostate cancer progression by promoting invasion, migration, and metastasis of prostate cancer by modulating gene expression changes, including modulation of *FAM101B*. Taken together, our work provides a better understanding of the function of NUSAP1 in aggressive prostate cancers, provides rationale for using *NUSAP1* as a prognostic biomarker, and sets the stage for developing improved therapeutic strategies for prostate and other cancers.

## MATERIALS AND METHODS

### Cell culture

Cell lines were grown under standard conditions, used by passage 15, and were originally purchased from ATCC (Manassas, VA, USA) with the exception of the PC-3-luc2 cell line, which was purchased from PerkinElmer (Waltham, MA, USA). DU145, DU145-NUSAP1, and HEK 293T cells were grown in DMEM (Life Technologies, Carlsbad, CA, USA). LNCaP cells were grown in T-Medium (Invitrogen, Carlsbad, CA, USA). PC-3 (ATCC), PC-3-luc2 (PerkinElmer), and PC-3-NUSAP1 cells were grown in F-12K Nutrient Mixture (Life Technologies). The 22Rv1 cell line was grown in RPMI-1640 Medium (Life Technologies).

### Lentiviral production

Lentiviral particles were produced as described previously [31].

### Lentiviral infections

Lentiviral infections were performed as described previously [31] to overexpress *NUSAP1* versus *EGFP* control, knockdown *NUSAP1* versus scramble control, or knockdown *FAM101B* versus scramble control when cells were 10 to 50% confluent. Antibiotics used for stable selection include puromycin (1-10 μg/ml) or hygromycin B (100-500 μg/ml).

### Plasmids

The pMDL, pRSV, and pMD2.G-VSVG plasmids were gifts from Dr. Julien Sage. The pLKO.1 puro vector with scramble shRNA sequence (Plasmid #1864) was purchased from Addgene (Cambridge, MA, USA) and deposited by Dr. David Sabatini [62]. Three pLKO.1 puro vectors with *NUSAP1* shRNA sequences (clone ID: TRCN0000135909 [NUSAP1 shRNA #1], clone ID: TRCN0000136422 [NUSAP1 shRNA #2], and clone ID: TRCN0000137707 [NUSAP1 shRNA #3]) were purchased from Thermo Fisher Scientific (Waltham, MA, USA). The pReceiver-Lv105 vector with *NUSAP1* cDNA (EX-Z29392-Lv105), pReceiver-Lv105 vector with *EGFP* cDNA (EX-EGFP-Lv105), and three psi-LVRU6MH vectors with *FAM101B* shRNA sequences (HSH009701-31-LVRU6MH [FAM101B shRNA #1] and HSH009701-32-LVRU6MH [FAM101B shRNA #2]) and scramble shRNA sequence (CSHCTR001-LVRU6MH) were purchased from GeneCopoeia (Rockville, MD, USA).

### RNA extraction and purification

Total RNA from cell lines, mouse tissues, and tumors was purified using TRIzol Reagent (Life Technologies) or RNeasy Microarray Tissue Mini Kit (Qiagen) as directed by the manufacturer's protocol. The RNeasy MinElute Cleanup Kit (Qiagen, Valencia, CA, USA) was used to cleanup RNA as directed by the manufacturer's protocol. RNA was subject to DNase treatment using the RNase-Free DNase Set (Qiagen) or TURBO DNA-free Kit (Life Technologies).

### RT-qPCR

RT-qPCR was performed as previously described [31]. Relative gene expression was determined with the ΔC_T_ method using *HPRT1* or universal *GAPDH* reference genes. Primer sequences are listed in Supplementary Table 4.

### RNA-Seq and expression analysis

Lentiviral infections were used to stably overexpress or knockdown *NUSAP1* or controls in triplicate in DU145 or PC-3 cell lines. After 72 or 96 hours, total RNA was extracted and purified. Sequencing libraries were prepared with the TruSeq RNA Sample Preparation Kit v2 (Illumina, San Diego, CA, USA) or TruSeq Stranded mRNA Sample Preparation Kit (Illumina) as directed by the manufacturer's protocol. Pooled libraries were run on a HiSeq 2000 Sequencing System (Illumina) with 101 base pair single-end reads. TopHat [63] and Cufflinks [64] software were used to align sequences to the human male (hg19) genome and determine expression levels, respectively. Differentially expressed genes had fold-change ≥ 1.5 and adjusted *P* < 0.05. Ingenuity Pathways Analysis (Qiagen) was used to identify biological functions associated with genes differentially expressed. Gene expression data have been deposited in Gene Expression Omnibus (GEO Accession: GSE80963).

### Measurements of cell cycle and apoptosis

Cells were harvested, fixed in 70% ethanol, stained with propidium iodide, and run on a BD Accuri C6 Flow Cytometer (BD Biosciences). DNA content from individual cells was analyzed using CFlow Sampler software (BD Biosciences), whereby the percentage of cells in G0/G1, G2/M, and apoptotic peaks was determined.

### Mouse xenograft assay and monitoring

Procedures with live animals were conducted in accordance with ethical standards and approved by Stanford University's Institutional Animal Care and Use Committee. Lentiviral infections were used to stably overexpress or knockdown *NUSAP1* or controls in PC-3-luc2 cells. Cells were harvested, counted using a hemocytometer, and resuspended in equal volumes of PBS and Matrigel (Corning Life Sciences, Corning, NY, USA). One million cells were subcutaneously injected into the left flank of isoflurane anesthetized 4 to 6 week old SCID-beige male mice (Charles River, Wilmington, MA, USA). Tumor volume was monitored weekly by bioluminescence imaging and caliper measurements. For bioluminescence imaging, luciferase expression was monitored with the IVIS 100 imaging system and Living Image software (PerkinElmer) by imaging mice 10 minutes post intraperitoneal injection of 150 mg/kg luciferin. Total flux was used to calculate tumor volume over time. For caliper measurements, the length (*l*), width (*w*), and height (*h*) of tumors were measured weekly with a caliper beginning 3 weeks after subcutaneous injection and tumor volume was calculated using the formula: *l* × *w* × *h* π ÷ 6.

After tumor volume monitoring (50, 59, or 62 days post injection of cells), mice were euthanized, and tissues and tumor biopsies were extracted and either immediately *ex vivo* imaged via bioluminescence imaging or saved for later RT-qPCR analysis. To save samples, tissues were stored in RNA*later* RNA Stabilization Reagent (Qiagen), Allprotect Tissue Reagent (Qiagen), or a -80°C freezer.

### Protein extraction and western blots

Protein extractions were performed and protein concentrations were determined as described previously [31]. Forty μg of total protein was loaded per lane on 4-20% Mini PROTEAN TGX Gels (Bio-Rad Laboratories, Hercules, CA, USA). Western blots were performed using anti-NUSAP1 (1:2,500; 12024-1-AP; Proteintech Group, Rosemont, IL, USA), anti-FAM101B (1:500; ab150350; Abcam, Cambridge, UK), or anti-GAPDH (1:4,000; 10R-G109A; Fitzgerald Industries International, Acton, MA, USA) primary antibodies; and Peroxidase AffiniPure Goat Anti-Mouse IgG, Light Chain Specific (1:10,000; 115-035-174 Jackson ImmunoResearch Laboratories, Inc., West Grove, PA, USA) or Peroxidase IgG Fraction Monoclonal Mouse Anti-Rabbit IgG, Light Chain Specific (1:5,000 or 1:10,000; 211-032-171; Jackson ImmunoResearch Laboratories, Inc.) secondary antibodies. Western blots were developed and stripped as described previously [31], and visualized using the Mini-Medical/90 film processor (AFP ImageWorks, Elmsford, NY, USA).

### Cell proliferation assays

Cell proliferation was quantified over 5 days using the CellTiter 96 Aqueous One Solution Cell Proliferation Assay (Promega, Madison, WI, USA) as directed by the manufacturer's protocol.

### Cell invasion assays

Invasion assays were performed with 100,000 DU145 cells overexpressing *NUSAP1* or control, 25,000 or 50,000 PC-3 cells overexpressing *NUSAP1* or control, or 50,000 or 125,000 PC-3-NUSAP1 cells underexpressing *FAM101B* or control using BD BioCoat Matrigel Invasion Chambers (BD Biosciences) as directed by the manufacturer's protocol with slight modifications as described previously [31]. Images were acquired using a QImaging QICAM digital camera (QImaging, Surrey, BC, Canada) using either a 4x or 10x objective on an Olympus IX51 inverted microscope (Olympus Corporation, Center Valley, PA, USA).

### Wound healing assays

Confluent monolayers of cells were abraded with a pipette tip and monitored for wound channel closure over time. Images were acquired using a QImaging QICAM digital camera (QImaging) using either a 4x or 10x objective on an Olympus IX51 inverted microscope (Olympus Corporation). NIH Image J software was used to measure wound channel area as a function of time.

### Data analysis

Microsoft Excel (Microsoft, Redmond, WA, USA), GraphPad Prism 6 (GraphPad Software, La Jolla, CA, USA), Java TreeView (version 1.1.6rv), SAM [40], and R (programming language) were used for analysis, to create graphs, or determine means, standard deviations, standard errors, and *P*-values (two-tailed Student's t-test, Mann-Whitney U test, Hypergeometric test, Log-rank test, Fisher's exact test).

## SUPPLEMENTARY FIGURES AND TABLES






