# Supplementary material for: NUSAP1 promotes invasion and metastasis of prostate cancer
Source: Oncotarget. 2017 Feb 22;8(18):29935–50. doi: 10.18632/oncotarget.15604 (PMC5444715; doi:10.18632/oncotarget.15604)
Supplement: Supplementary file 1 [file oncotarget-08-29935-s001.pdf]

# NUSAP1 promotes invasion and metastasis of prostate cancer

## SUPPLEMENTARY FIGURES AND TABLES

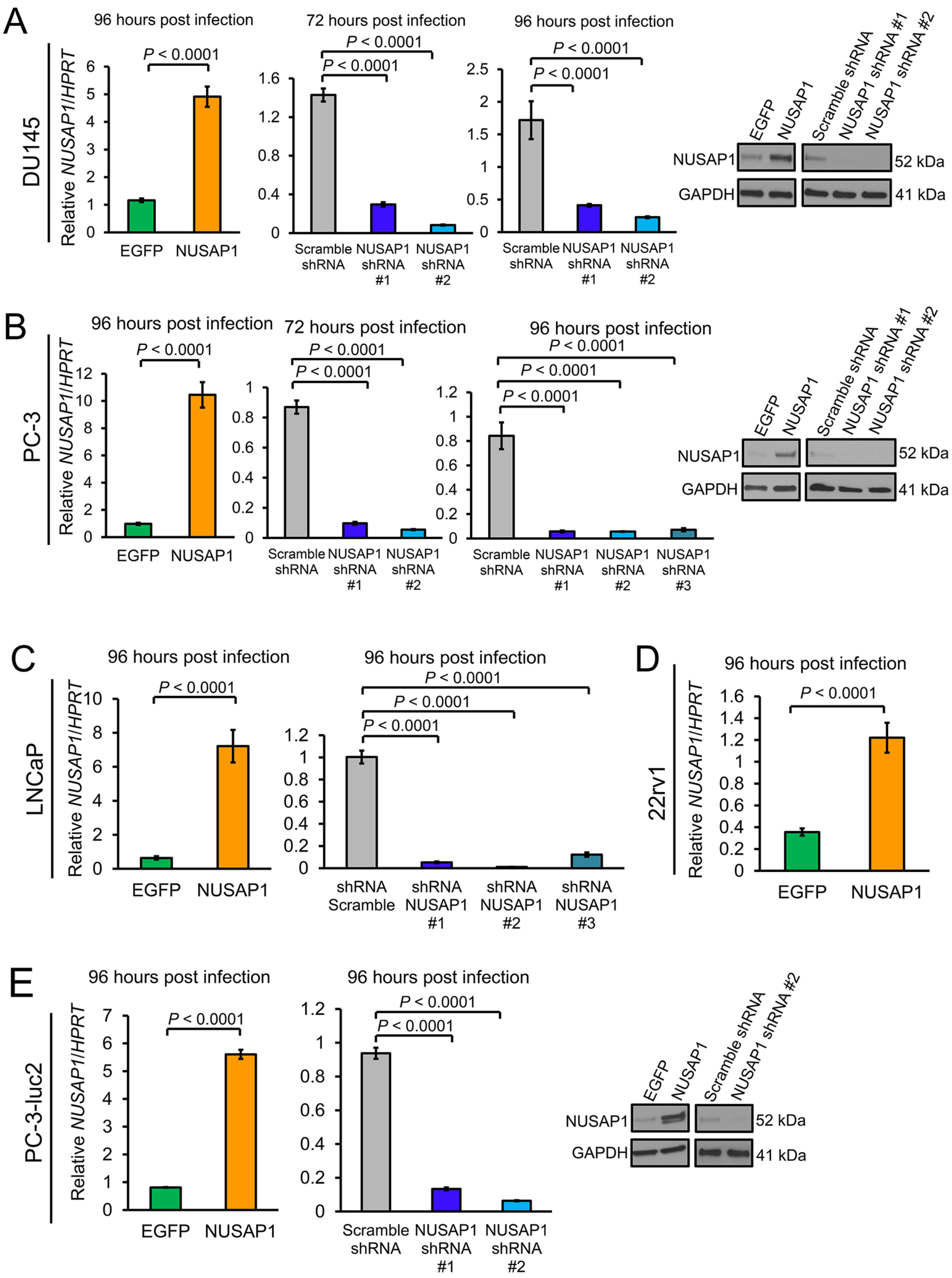

**Supplementary Figure 1: *NUSAP1* expression levels in cells overexpressing or underexpressing *NUSAP1*.** Lentiviral infections were used to overexpress or knockdown *NUSAP1* or controls in **A.** DU145, **B.** PC-3, **C.** LNCaP, **D.** 22Rv1 cells, or **E.** PC-3-luc2 cells. RNA was extracted from cells at indicated times post lentiviral infections and RT-qPCR was performed using SYBR green chemistry. Relative *NUSAP1* expression was determined by normalization to the *HPRT1* reference gene. Bars: mean ± SD (standard deviation). *P*-values were calculated using the two-tailed Student's t-test. Proteins were extracted 96 hours post lentiviral infections and western blots were performed with antibodies recognizing NUSAP1 and GAPDH.

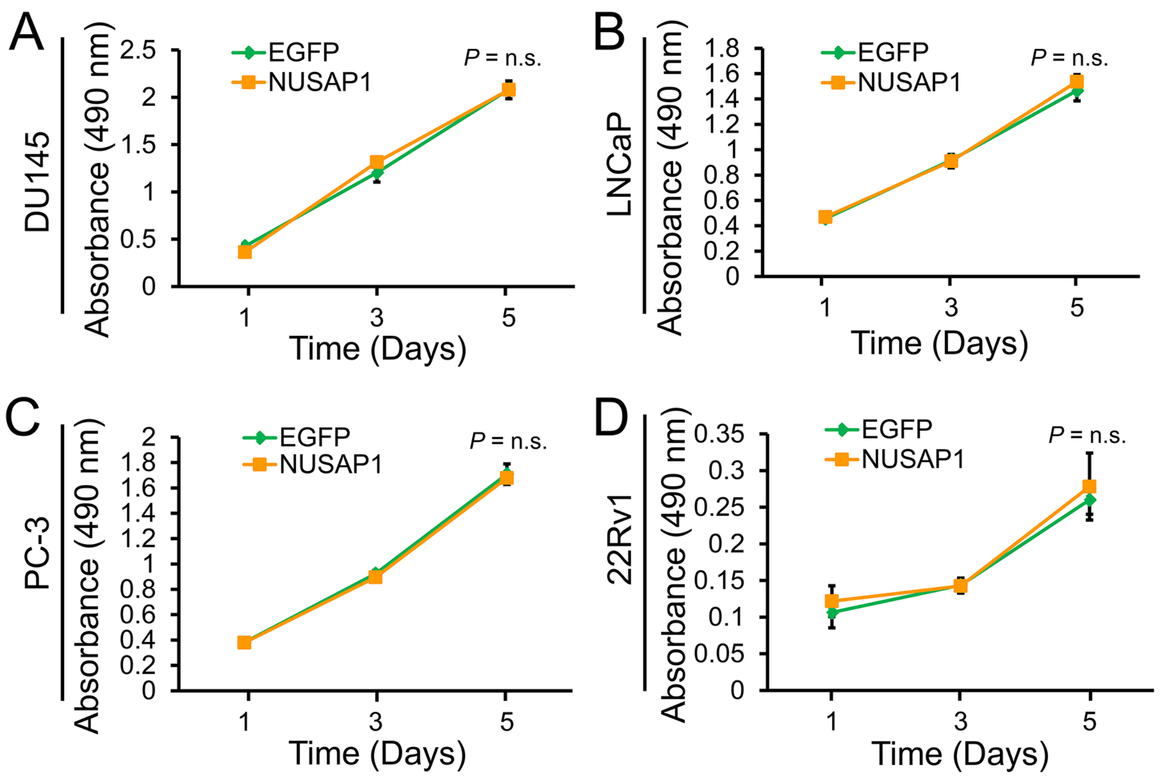


**Supplementary Figure 2: *NUSAP1* overexpression does not affect proliferation of prostate cancer cells grown in culture.** Lentiviral infections were used to stably overexpress *NUSAP1* or *EGFP* control in **A.** DU145, **B.** LNCaP, **C.** PC-3, or **D.** 22Rv1 prostate cancer cell lines. Cells were assessed for proliferation over 5 days using the CellTiter 96 Aqueous One Solution Cell Proliferation Assay. Points: mean ± SD. *P*-values were calculated using the two-tailed Student's t-test.

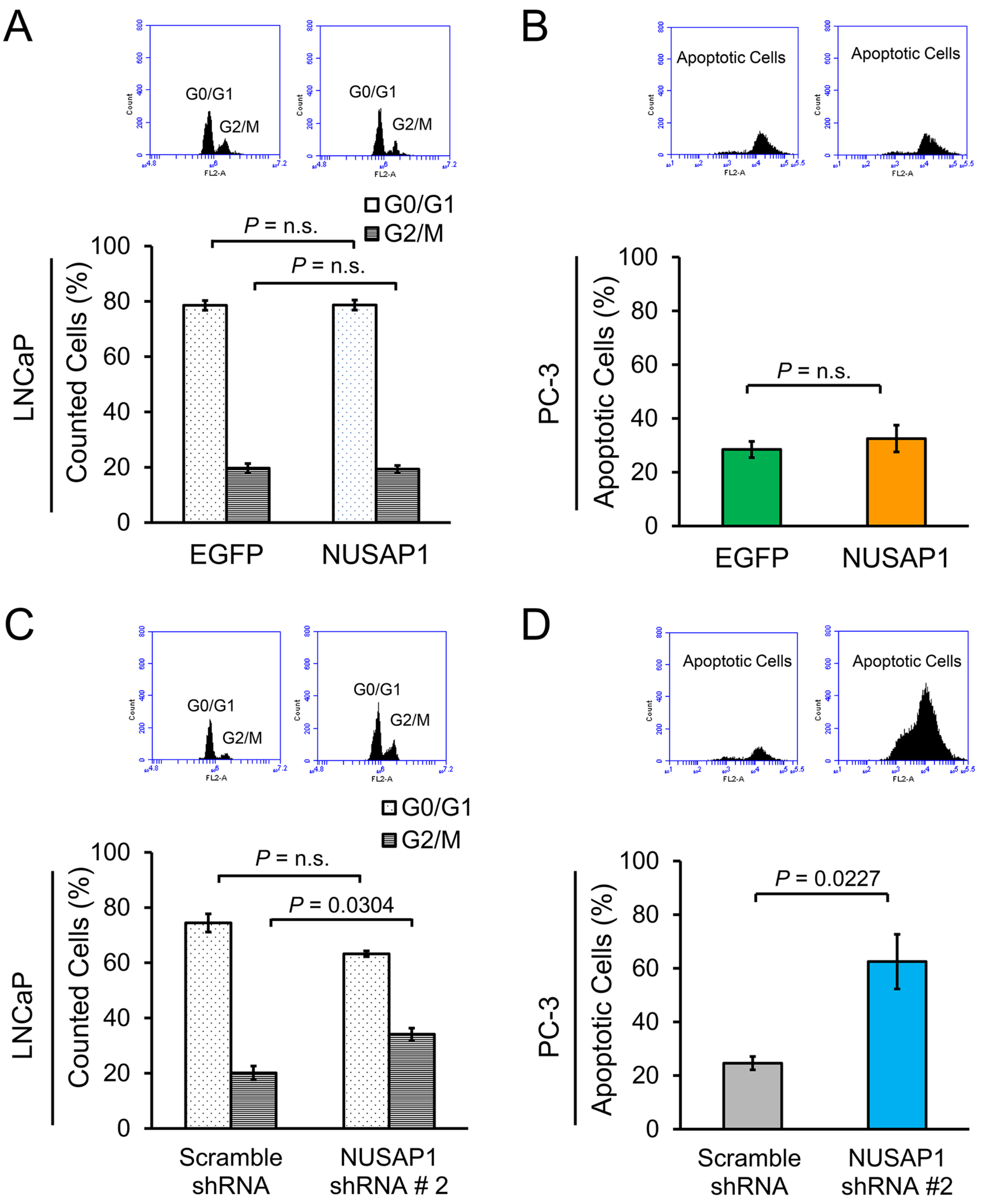


**Supplementary Figure 3: Analysis of G0/G1, G2/M, and apoptotic cells upon *NUSAP1* overexpression or knockdown.** Prostate cancer cells overexpressing or underexpressing *NUSAP1* or controls were stained with propidium iodide for flow cytometry analysis. **A.** G0/G1 and G2/M distribution analysis of LNCaP cells with *NUSAP1* overexpression. **B.** Apoptotic distribution analysis of PC-3 cells with *NUSAP1* overexpression. **C.** G0/G1 and G2/M distribution analysis of LNCaP cells with *NUSAP1* knockdown. **D.** Apoptotic distribution analysis of PC-3 cells with *NUSAP1* knockdown. All images represent original flow cytometry data. All bars: mean ± SEM. All *P*-values were calculated using the two-tailed Student's t-test.

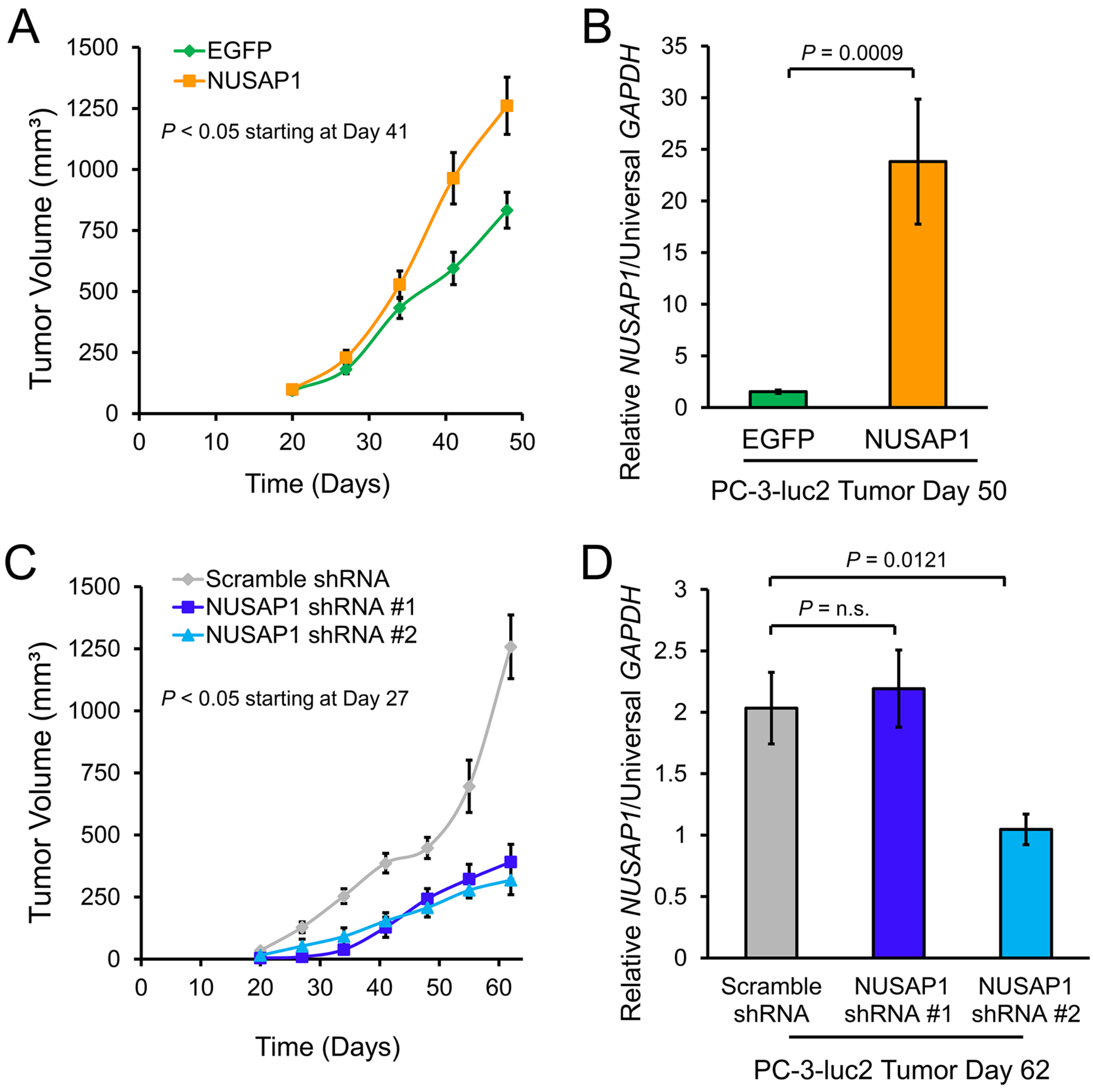


**Supplementary Figure 4: *NUSAP1* overexpression eventually increases tumor volume while *NUSAP1* knockdown decreases tumor volume.** PC-3-luc2 cells overexpressing or underexpressing *NUSAP1* or controls were subcutaneously injected into the flanks of male SCID-beige mice. **A.** Caliper measurements of tumor volume over time in mice with tumors overexpressing *NUSAP1* versus *EGFP*. Points: mean ± SEM. EGFP: n = 21; NUSAP: n = 21. **B.** RNA was harvested from tumors overexpressing *NUSAP1* or *EGFP* at day 50 and RT-qPCR was performed using SYBR green chemistry. Relative *NUSAP1* expression was determined by normalization to the universal *GAPDH* reference gene. Bars: mean ± SD. EGFP: n = 12; NUSAP1: n = 11. **C.** Caliper measurements of tumor volume over time in mice with tumors underexpressing *NUSAP1* versus control. Points: mean ± SEM. Scramble shRNA: n = 10; NUSAP1 shRNA #1: n = 11; NUSAP1 shRNA #2: n = 7. **D.** RNA was harvested from tumors with knockdown of *NUSAP1* or control at day 62 and RT-qPCR was performed using SYBR green chemistry. Relative *NUSAP1* expression was determined by normalization to the universal *GAPDH* reference gene. Bars: mean ± SD. Scramble shRNA: n = 12; NUSAP1 shRNA # 1: n = 9; NUSAP1 shRNA #2: n = 9. All *P*-values were calculated using the two-tailed Student's t-test.

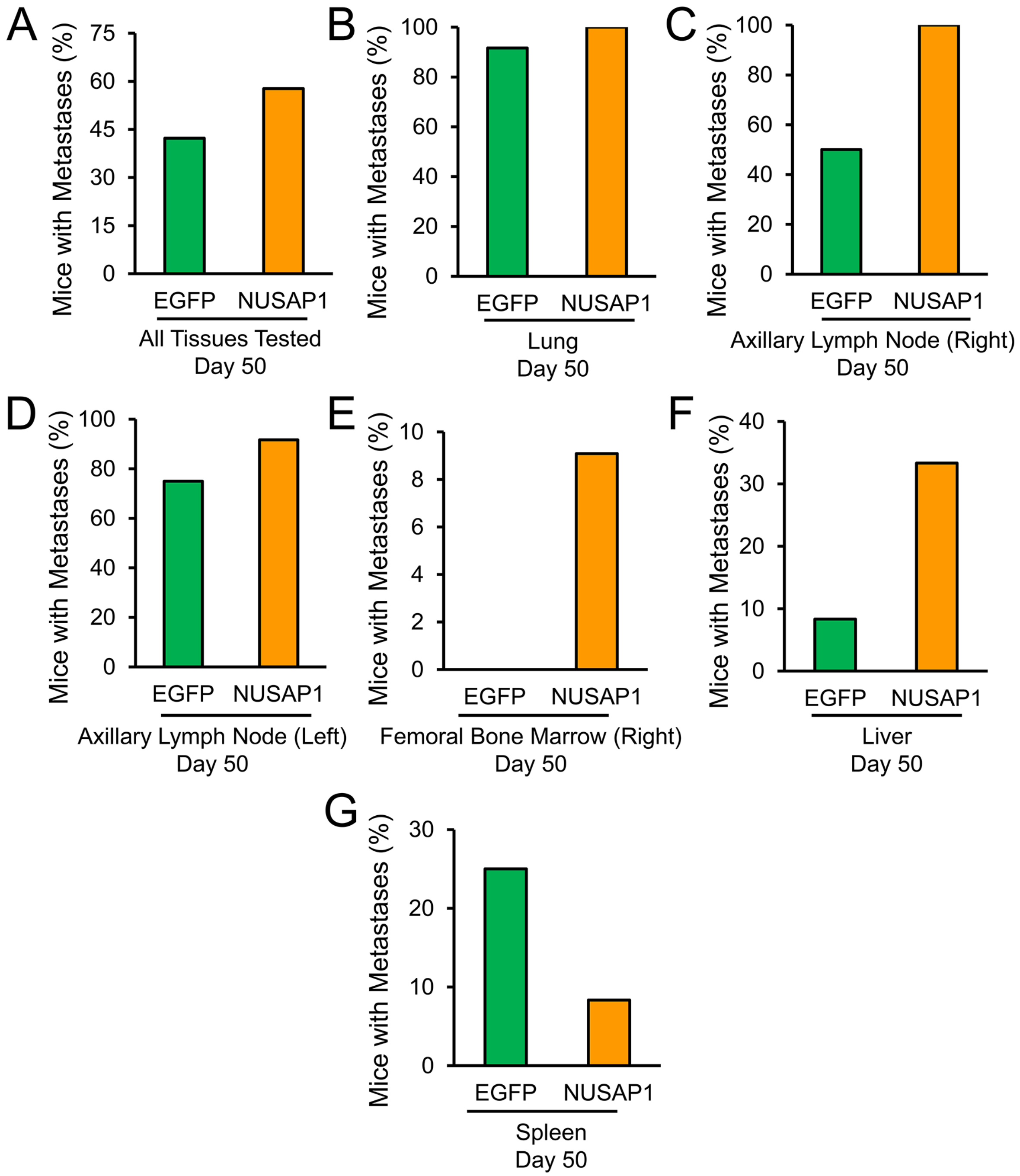


**Supplementary Figure 5: Percentage of mice with metastases is generally higher when *NUSAP1* is overexpressed in the flank tumor.** PC-3-luc2 cells overexpressing *NUSAP1* or *EGFP* control were subcutaneously injected into the flanks of male SCID-beige mice. On day 50, mice were sacrificed and tissues (lungs, axillary lymph nodes [right and left], femoral bone marrow [right], livers, and spleens) were extracted to quantify metastases by RT-qPCR using SYBR green chemistry. Expression of human-specific *GAPDH* relative to universal *GAPDH* was used to quantify metastases, and percentage of mice with metastases was determined for **A.** all six tissue sites combined and the **B.** lung, **C.** right axillary lymph node, **D.** left axillary lymph node, **E.** right femoral bone marrow, **F.** liver, and **G.** spleen.

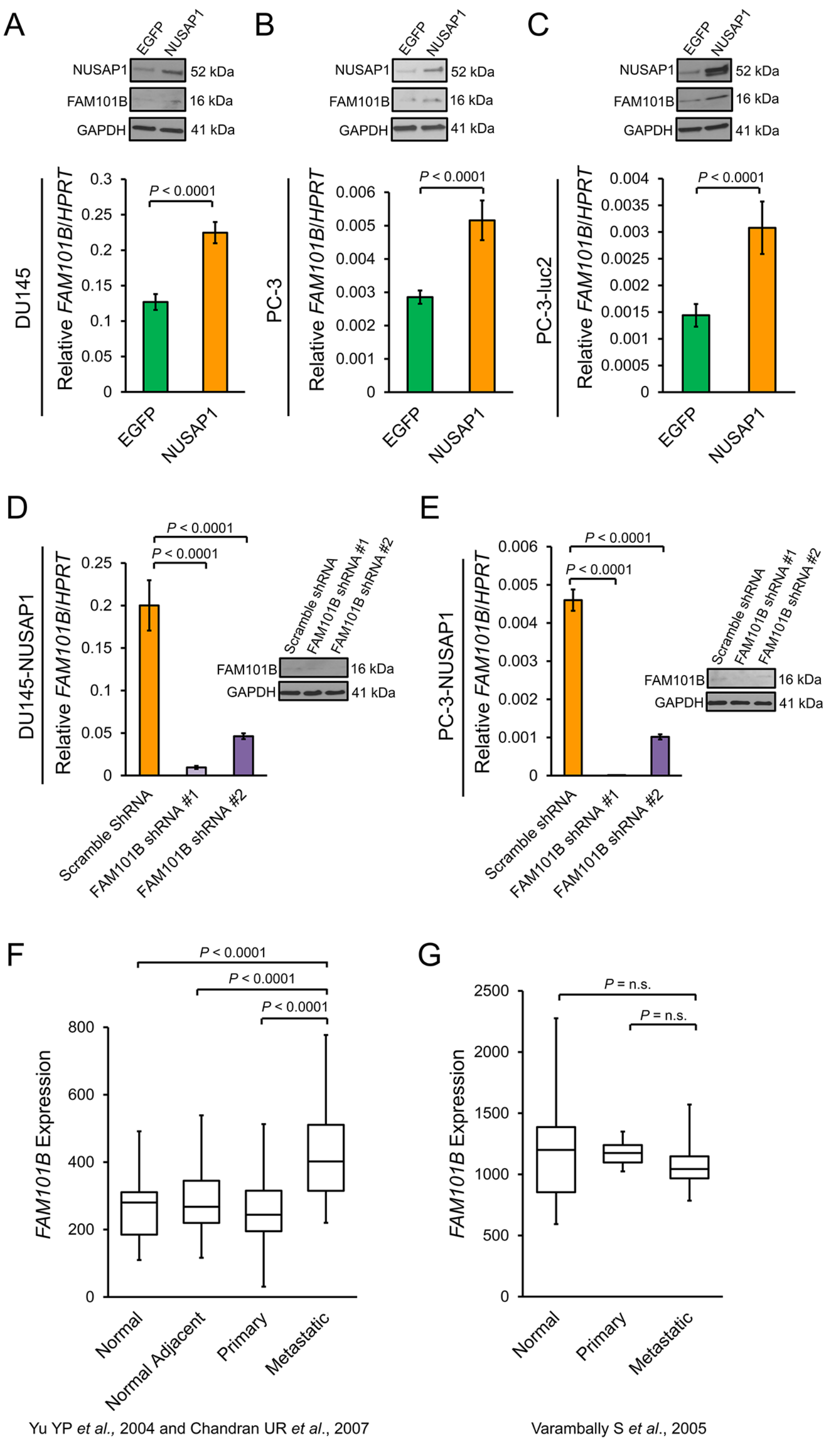


**Supplementary Figure 6: *FAM101B* expression in prostate cancer cell lines and patient samples.** **A-E.** Lentiviral infections were used to stably overexpress *NUSAP1* or knockdown *FAM101B* or controls in (A) DU145, (B) PC-3, (C) PC-3-luc2 cells, (D) DU145-NUSAP1, or (E) PC-3-NUSAP1 cells. RNA and proteins were extracted from cells 96 hours post lentiviral infections. RT-qPCR was performed using SYBR green chemistry. Relative *FAM101B* expression was determined by normalization to the *HPRT1* reference gene. Bars: mean ± SD. Western blots were performed with antibodies recognizing NUSAP1, FAM101B, and GAPDH. **F.** Box plots of *FAM101B* expression in the Yu YP *et al.*, 2004 [36] and Chandran UR *et al.*, 2007 [37] datasets (GEO Accession: GDS2546; GEO Profile: 34877214). Normal: n = 17; Normal Adjacent: n = 59; Primary: n = 66; Metastatic: n = 25. **G.** Box plots of *FAM101B* expression in the Varambally S *et al.*, 2005 [38] dataset (GEO Accession: GDS1439; GEO Profile: 14261532). Normal: n = 6; Primary: n = 7; Metastatic: n = 6. All *P*-values were calculated using the two-tailed Student's t-test.

**Supplementary Table 1: Metastases as seen by *ex vivo* bioluminescence imaging**

| Day | Expression | Metastases | | | | | |
|---|---|---|---|---|---|---|---|
| | | Lung | | Right Axillary Lymph Node | | Left Axillary Lymph Node | |
| | | number | % | number | % | number | % |
| 50 | EGFP | 0/5 | 0 | 1/5 | 20 | 0/5 | 0 |
| 50 | NUSAP1 | 2/6 | 33.3 | 3/6 | 50 | 2/6 | 33.3 |
| 59 | EGFP | 3/6 | 60 | 2/6 | 33.3 | 2/6 | 33.3 |
| 59 | NUSAP1 | 4/6 | 66.7 | 5/6 | 83.3 | 3/6 | 50 |
| 62 | Scramble shRNA | 3/6 | 50 | 2/6 | 33.3 | 2/6 | 33.3 |
| 62 | NUSAP1 shRNA #1 | 1/6 | 16.7 | 0/6 | 0 | 1/6 | 16.7 |
| 62 | NUSAP1 shRNA #2 | 0/7 | 0 | 2/7 | 28.6 | 0/7 | 0 |

**Supplementary Table 2: Differentially expressed genes and their fold-changes as determined by RNA-Seq when *NUSAP1* is overexpressed in PC-3 cells.**

**See Supplementary File 1**

**Supplementary Table 3: Differentially expressed genes and their fold-changes as determined by RNA-Seq when *NUSAP1* is knocked down in DU145 and PC-3 cells.**

**See Supplementary File 2**

**Supplementary Table 4: Primer sequences used for RT-qPCR**

| Gene | Primer Sequence |
|---|---|
| *NUSAP1* | Forward: 5'-CCCTCAAGTACAGTGACCTGC-3'<br>Reverse: 5'-TCATTTCCTTTTCTTGCCTCA-3' |
| *HPRT1* | Forward: 5'-TGACACTGGCAAAACAATGCA-3'<br>Reverse: 5'-GGTCCTTTTCACCAGCAAGCT-3 |
| Human-specific *GAPDH* | Forward: 5'-AGATCCCTCCAAAATCAAGTG-3'<br>Reverse: used universal *GAPDH* reverse primer |
| Universal *GAPDH* | Forward: 5'-CCATGGAGAAGGCTGGGG-3'<br>Reverse: 5'-CAAAGTTGTCATGGATGACC-3' |
| *FAM101B* | Forward: 5'-CTGAGGCTTTGTCCCCTGT-3'<br>Reverse: 5'-AAGTGCCTCTCGGAGTCGTA-3' |
