# Supplementary material for: NUSAP1 promotes invasion and metastasis of prostate cancer
Source: Oncotarget. 2017 Feb 22;8(18):29935–50. doi: 10.18632/oncotarget.15604 (PMC5444715; doi:10.18632/oncotarget.15604)
Supplement: Supplementary file 2 [file oncotarget-08-29935-s002.docx]

**Supplementary Table S2: Differentially expressed genes and their fold-changes as determined by RNA-Seq when *NUSAP1* is overexpressed in PC-3 cells.**

|  | **Gene Symbol** | **Fold-change** |
| --- | --- | --- |
| 1 | ABCA3 | 1.5 |
| 2 | ADAM12 | 1.5 |
| 3 | ADAM21 | -1.6 |
| 4 | ADAMTS1 | -2.3 |
| 5 | AMACR | 1.5 |
| 6 | ANKRD1 | -1.5 |
| 7 | ANKRD12 | -1.5 |
| 8 | ANKRD20A5P | -1.5 |
| 9 | ANXA2R | -1.5 |
| 10 | APCDD1L | 2.4 |
| 11 | APLN | 1.7 |
| 12 | APOLD1 | 1.5 |
| 13 | ASIC3 | 1.8 |
| 14 | ATAT1 | -1.5 |
| 15 | ATF3 | -1.5 |
| 16 | C11orf91 | -3.4 |
| 17 | C16orf46 | -1.8 |
| 18 | C17orf104 | -1.8 |
| 19 | C17orf67 | 1.6 |
| 20 | C1orf233 | 1.7 |
| 21 | C1S | -2.4 |
| 22 | C9orf69 | 1.8 |
| 23 | CAB39L | -1.6 |
| 24 | CACNB1 | -1.5 |
| 25 | CALCOCO1 | -1.5 |
| 26 | CAMK1G | -7 |
| 27 | CCDC85B | -1.5 |
| 28 | CCL26 | -3.9 |
| 29 | CLDN11 | 1.7 |
| 30 | CPA4 | -1.5 |
| 31 | CPT2 | 1.6 |
| 32 | CST4 | 2.2 |
| 33 | CTGF | -3.7 |
| 34 | CTNND2 | 1.7 |
| 35 | CTSK | 1.5 |
| 36 | CXCL12 | 1.9 |
| 37 | CYR61 | -1.8 |
| 38 | DAW1 | -1.9 |
| 39 | DBNDD2 | -1.9 |
| 40 | DGCR14 | -1.5 |
| 41 | DMBX1 | -1.9 |
| 42 | DNAJC30 | 1.5 |
| 43 | DOCK8 | 1.7 |
| 44 | DUSP1 | -1.9 |
| 45 | EPB41L4A | -1.5 |
| 46 | EXOC2 | 1.6 |
| 47 | FAM101B | 1.6 |
| 48 | FAM86JP | 1.6 |
| 49 | FAM89A | 1.7 |
| 50 | FBXO4 | 1.5 |
| 51 | FCGR1A | -5.5 |
| 52 | FCGR1B | -3.5 |
| 53 | FCGR2A | -2.2 |
| 54 | FLJ42627 | -1.5 |
| 55 | FOXD2-AS1 | -1.7 |
| 56 | FOXD4 | -2.6 |
| 57 | FOXD4L1 | -4.4 |
| 58 | FTCD | -3.8 |
| 59 | GALK1 | 1.7 |
| 60 | GALNT12 | 1.5 |
| 61 | GCNT3 | -4 |
| 62 | GDF15 | -1.6 |
| 63 | GMEB1 | -1.5 |
| 64 | GNAZ | 1.5 |
| 65 | GRB7 | -1.5 |
| 66 | H6PD | 2.1 |
| 67 | HADH | 1.7 |
| 68 | HEBP1 | 1.8 |
| 69 | HIST2H4A | -1.6 |
| 70 | HLA-F-AS1 | -1.7 |
| 71 | HLX | 1.5 |
| 72 | HOTAIRM1 | -1.5 |
| 73 | HOXA2 | -3.1 |
| 74 | HOXA5 | -1.5 |
| 75 | HSH2D | -1.7 |
| 76 | HSPA1A | -1.9 |
| 77 | HSPA1B | -2 |
| 78 | HSPA6 | -3.7 |
| 79 | HSPA7 | -2.3 |
| 80 | HTRA1 | 1.8 |
| 81 | IER3 | -1.5 |
| 82 | IL24 | -2.1 |
| 83 | JAK3 | 1.5 |
| 84 | JOSD2 | 1.5 |
| 85 | JUNB | -1.7 |
| 86 | KCNK15 | 2.4 |
| 87 | KCNK7 | -2.8 |
| 88 | KCTD11 | 1.5 |
| 89 | KIAA1024 | -1.5 |
| 90 | KIAA1875 | -2.5 |
| 91 | KRT85 | -1.6 |
| 92 | KRTAP2-3 | -1.7 |
| 93 | LAT | -1.7 |
| 94 | LILRA6 | -3.4 |
| 95 | LINC00641 | -1.6 |
| 96 | LMF1 | 1.9 |
| 97 | LOC100288181 | 1.9 |
| 98 | LOC100288911 | -1.5 |
| 99 | LOC100506136 | -3.6 |
| 100 | LOC100507053 | -1.6 |
| 101 | LOC153684 | -1.5 |
| 102 | LOC284837 | -3.5 |
| 103 | LOC339666 | -2.5 |
| 104 | LOC401320 | -1.5 |
| 105 | LOC729013 | 1.6 |
| 106 | LOC90834 | -1.9 |
| 107 | LOXL2 | 1.8 |
| 108 | LYPD3 | -1.8 |
| 109 | MAGEB17 | -2.5 |
| 110 | MAP3K10 | 1.5 |
| 111 | MGC16121 | -2.5 |
| 112 | MGC21881 | 1.5 |
| 113 | MGMT | 1.5 |
| 114 | MIR100HG | -1.6 |
| 115 | MIRLET7DHG | -1.5 |
| 116 | MMP13 | 1.7 |
| 117 | MT2A | -1.6 |
| 118 | MTSS1 | -1.6 |
| 119 | NAGLU | 1.6 |
| 120 | NAGS | 1.5 |
| 121 | NAP1L2 | -1.6 |
| 122 | NAPRT1 | -1.5 |
| 123 | NEAT1 | -1.9 |
| 124 | NLRC4 | -1.5 |
| 125 | NMNAT3 | 1.9 |
| 126 | NODAL | -1.7 |
| 127 | NT5M | 2.1 |
| 128 | NUSAP1 | 3.1 |
| 129 | OBSL1 | 1.8 |
| 130 | OPRL1 | 1.5 |
| 131 | OSBPL7 | -1.5 |
| 132 | OSCP1 | 2.6 |
| 133 | OXLD1 | 1.7 |
| 134 | PANK2 | -1.5 |
| 135 | PAPLN | -2.1 |
| 136 | PARVB | 1.6 |
| 137 | PDLIM4 | 1.6 |
| 138 | PGLS | 1.9 |
| 139 | PHACTR3 | -1.7 |
| 140 | PI4K2B | 1.7 |
| 141 | PLCH2 | 2.5 |
| 142 | PPAPDC2 | 1.5 |
| 143 | PSMG4 | 1.5 |
| 144 | PTGES | -1.6 |
| 145 | RASD1 | -1.9 |
| 146 | REEP6 | 1.5 |
| 147 | RHEBL1 | -1.5 |
| 148 | ROBO4 | 1.5 |
| 149 | RSAD2 | -1.5 |
| 150 | SAT1 | -2.3 |
| 151 | SCEL | -1.6 |
| 152 | SCNN1G | 1.7 |
| 153 | SCUBE3 | 1.7 |
| 154 | SEC14L1P1 | -2.1 |
| 155 | SEC31B | -2.6 |
| 156 | SEPT3 | 1.6 |
| 157 | SERPINA1 | 1.6 |
| 158 | SGK196 | -1.6 |
| 159 | SIRPB1 | 1.6 |
| 160 | SLC25A34 | -3.9 |
| 161 | SNHG3 | -1.8 |
| 162 | SPATA7 | 1.6 |
| 163 | SRD5A3 | 1.7 |
| 164 | STC1 | 1.5 |
| 165 | SYT11 | -1.5 |
| 166 | TAS2R5 | -2.6 |
| 167 | THAP10 | 1.7 |
| 168 | TLR6 | -1.5 |
| 169 | TMEM120B | -1.5 |
| 170 | TMEM132A | 1.5 |
| 171 | TMEM158 | 1.5 |
| 172 | TNFSF9 | -1.9 |
| 173 | TREX1 | -1.6 |
| 174 | TRIM65 | 1.6 |
| 175 | TTC32 | -1.5 |
| 176 | TUBB1 | -2 |
| 177 | TXK | -1.7 |
| 178 | TYSND1 | 1.5 |
| 179 | UROS | 1.6 |
| 180 | WWC3 | 2 |
| 181 | XK | 1.5 |
| 182 | ZNF132 | -2.4 |
| 183 | ZNF469 | 1.6 |
| 184 | ZNF487P | -1.9 |
| 185 | ZNF713 | -1.6 |
