# Supplementary material for: NUSAP1 promotes invasion and metastasis of prostate cancer
Source: Oncotarget. 2017 Feb 22;8(18):29935–50. doi: 10.18632/oncotarget.15604 (PMC5444715; doi:10.18632/oncotarget.15604)
Supplement: Supplementary file 3 [file oncotarget-08-29935-s003.docx]

**Supplementary Table S3: Differentially expressed genes and their fold-changes as determined by RNA-Seq when *NUSAP1* is knocked down in DU145 and PC-3 cells.**

|  | **Gene Symbol** | **Fold-change** | | | | | | | |
| --- | --- | --- | --- | --- | --- | --- | --- | --- | --- |
|  |  | **DU145** | | | | **PC-3** | | | |
|  |  | **NUSAP1 shRNA** | | | | **NUSAP1 shRNA** | | | |
|  |  | **#1** | | **#2** | | **#1** | | **#2** | |
|  |  | **72 hrs** | **96 hrs** | **72 hrs** | **96 hrs** | **72 hrs** | **96 hrs** | **72 hrs** | **96 hrs** |
| 1 | ABCE1 | -1.8 | -2 | -1.8 | -2.6 | -2 | -2.1 | -1.7 | -1.6 |
| 2 | ABLIM1 | -2.9 | -2.9 | -3 | -3.4 | -2.3 | -2.1 | -1.7 | -1.9 |
| 3 | AKT3 | 1.7 | 2.5 | 1.5 | 2.6 | 1.7 | 1.7 | 2.4 | 2 |
| 4 | ALDH1A3 | -1.7 | -2 | -3.9 | -8.1 | -2.5 | -1.9 | -7.4 | -3.6 |
| 5 | ALDH7A1 | -1.5 | -1.8 | -2 | -3.8 | -2.1 | -1.5 | -2.7 | -3.8 |
| 6 | ALDH9A1 | -2 | -1.8 | -2.2 | -2.6 | -2.3 | -2.3 | -1.5 | -2.3 |
| 7 | ANP32E | -1.5 | -1.7 | -1.6 | -2.3 | -2.3 | -3 | -1.9 | -2.5 |
| 8 | ARL4C | 3.9 | 5.2 | 3.5 | 8.7 | 2.2 | 1.5 | 2.1 | 1.9 |
| 9 | BTG2 | 2.5 | 2.2 | 2.6 | 2.8 | 2.8 | 2.6 | 3 | 3.6 |
| 10 | C10orf12 | -1.8 | -2.1 | -1.8 | -2 | -3.1 | -3.5 | -1.9 | -2.1 |
| 11 | C20orf112 | 2.6 | 3.7 | 2.8 | 5.6 | 2.1 | 1.9 | 3.5 | 3.7 |
| 12 | C6orf141 | 5 | 5.2 | 10.7 | 25.5 | 2.9 | 2.5 | 4.2 | 8.7 |
| 13 | CACNB3 | 2.1 | 2.1 | 2.4 | 3.2 | 2.2 | 1.7 | 2.1 | 2 |
| 14 | CBX5 | -1.6 | -1.5 | -2.8 | -3.8 | -2.1 | -2.6 | -3.3 | -5.6 |
| 15 | CCSAP | -2.4 | -2.3 | -2.3 | -2 | -2.1 | -1.9 | -1.7 | -2.3 |
| 16 | CD68 | 3.3 | 4.6 | 2.8 | 7.6 | 2 | 3.9 | 2.2 | 3.8 |
| 17 | CDK5RAP2 | -1.9 | -2.3 | -1.6 | -1.8 | -2.2 | -2.2 | -2 | -2.5 |
| 18 | CELSR3 | 1.5 | 1.5 | 2.5 | 3.3 | 1.9 | 2.7 | 2.2 | 4.1 |
| 19 | CENPE | -1.5 | -1.8 | -2.6 | -6.7 | -5.4 | -10.2 | -3.6 | -6.2 |
| 20 | CENPF | -1.5 | -1.9 | -2.3 | -4.9 | -4.4 | -7.6 | -3.1 | -6.2 |
| 21 | CHML | -1.9 | -1.7 | -7.9 | -11.9 | -2.7 | -2 | -4.7 | -6.3 |
| 22 | CKAP2 | -1.6 | -1.6 | -1.7 | -2.1 | -2.8 | -3.5 | -1.8 | -3.3 |
| 23 | CLP1 | -2.9 | -2.8 | -2.7 | -3.1 | -2.5 | -2.5 | -1.9 | -2.1 |
| 24 | CMTM3 | 4 | 3.9 | 2.6 | 2.6 | 3.3 | 3.4 | 5 | 6.2 |
| 25 | CNRIP1 | 2.3 | 3 | 1.5 | 2.7 | 3 | 2.6 | 2.2 | 2.1 |
| 26 | CRISPLD1 | 1.8 | 1.8 | 1.7 | 1.6 | 4.1 | 6.2 | 2.8 | 3.1 |
| 27 | CSRNP1 | 4.6 | 2.8 | 3.3 | 5.6 | 2.6 | 3.1 | 2 | 2.7 |
| 28 | CUL4A | -2.4 | -2.4 | -2.9 | -4.2 | -2.2 | -2.7 | -1.7 | -1.9 |
| 29 | CWF19L2 | -1.6 | -1.7 | -1.8 | -2.3 | -2.4 | -2.9 | -1.7 | -1.8 |
| 30 | DCAF12 | -2.6 | -2.7 | -3.6 | -3.1 | -3.1 | -3 | -2.1 | -2.4 |
| 31 | DDAH1 | -3.9 | -3.1 | -2.5 | -2.6 | -5.9 | -4.4 | -4.6 | -5.6 |
| 32 | DHTKD1 | -1.7 | -1.8 | -1.6 | -1.7 | -2.5 | -3 | -1.6 | -3.2 |
| 33 | DHX9 | -2 | -2.6 | -2.1 | -3.2 | -2.5 | -3 | -1.9 | -2.2 |
| 34 | DISP2 | 2.9 | 1.7 | 10.5 | 10.6 | 2.4 | 2 | 4.7 | 5.7 |
| 35 | DLGAP5 | -1.6 | -1.8 | -1.9 | -2.9 | -3.5 | -6.1 | -1.6 | -4.5 |
| 36 | DNAJC21 | -1.9 | -1.9 | -3.7 | -4 | -2.7 | -2 | -2.7 | -2.4 |
| 37 | DNAJC8 | -1.7 | -2.1 | -2.2 | -3.6 | -2 | -2.4 | -2.1 | -2.8 |
| 38 | DUSP16 | 1.5 | 2.3 | 1.9 | 3.4 | 2.3 | 5.2 | 1.7 | 3.3 |
| 39 | DYNLRB1 | 2.1 | 1.7 | 1.9 | 2.3 | 2.6 | 5.3 | 3.2 | 5 |
| 40 | EFNB3 | 1.6 | 2.7 | 3.1 | 6.9 | 1.8 | 2 | 2.4 | 1.7 |
| 41 | EIF4H | -1.7 | -2.3 | -1.7 | -3.1 | -1.7 | -2.4 | -2 | -2.4 |
| 42 | ENTPD5 | -2.9 | -2.6 | -2.2 | -2.6 | -2.9 | -2.3 | -2 | -2.4 |
| 43 | FAM101B | -1.5 | -3.2 | -1.7 | -5 | -2.6 | -7.4 | -4.7 | -8.2 |
| 44 | FAM208A | -1.8 | -1.6 | -2.7 | -2.2 | -2 | -1.6 | -2.6 | -2.4 |
| 45 | FEZ2 | -1.8 | -2 | -1.9 | -2.3 | -1.6 | -1.6 | -3.5 | -4.4 |
| 46 | FGFR1OP | -1.8 | -2.3 | -2 | -2.3 | -2.6 | -1.8 | -2.1 | -2.2 |
| 47 | GHR | 1.8 | 2.5 | 1.9 | 2.9 | 1.7 | 2.6 | 2.3 | 2.1 |
| 48 | GNG2 | 2 | 4.2 | 1.9 | 7.6 | 2.8 | 3.1 | 2.2 | 1.7 |
| 49 | GRK5 | 1.8 | 2.1 | 1.8 | 2 | 2.3 | 2.8 | 2.1 | 1.7 |
| 50 | HMGN2 | -1.5 | -1.9 | -1.6 | -2.5 | -1.8 | -2.2 | -2.1 | -3.5 |
| 51 | HNRNPM | -3 | -4 | -1.7 | -2.7 | -2.2 | -4.9 | -1.5 | -2.4 |
| 52 | HNRNPUL2 | -1.6 | -1.8 | -2.2 | -2.9 | -2 | -1.7 | -2.3 | -2.5 |
| 53 | HSD17B10 | 2.5 | 1.5 | 2.6 | 1.6 | 3.8 | 3.5 | 3 | 3 |
| 54 | IFI44 | 2.3 | 10.1 | 5.9 | 130.5 | 6.9 | 19 | 2.8 | 6.3 |
| 55 | IK | -2.4 | -2.5 | -2 | -2.5 | -2.3 | -2.5 | -1.8 | -2.3 |
| 56 | KIF15 | -2.2 | -3 | -2.1 | -3.5 | -3.6 | -4.3 | -3 | -5.3 |
| 57 | KIF23 | -2.1 | -3 | -1.7 | -2.6 | -4.7 | -6.9 | -2.2 | -3.2 |
| 58 | KPNB1 | -1.5 | -1.7 | -2.5 | -3.1 | -1.5 | -1.7 | -2.3 | -2.6 |
| 59 | LGALS3BP | 1.7 | 2.7 | 7.7 | 36.9 | 6.3 | 16 | 6.2 | 8.7 |
| 60 | LIMK1 | 3 | 1.6 | 2.3 | 1.8 | 2.3 | 1.5 | 1.7 | 2.3 |
| 61 | LIN7C | -2.6 | -2.6 | -2.3 | -2.4 | -2.8 | -2 | -1.9 | -1.6 |
| 62 | LIN9 | -1.6 | -2 | -1.7 | -2.2 | -2.5 | -3.4 | -1.7 | -2.8 |
| 63 | LMAN1 | -2.4 | -3 | -1.7 | -2.4 | -3.9 | -3.6 | -2.6 | -2.6 |
| 64 | LPPR2 | 2.2 | 1.7 | 5 | 5 | 2.1 | 2.5 | 3.1 | 4.1 |
| 65 | LYAR | -1.9 | -3.3 | -1.6 | -2.6 | -2.1 | -4.2 | -1.8 | -2.6 |
| 66 | LZTFL1 | -3.2 | -3.1 | -4 | -5.1 | -3.1 | -2.4 | -2.1 | -1.6 |
| 67 | MAMLD1 | 4.3 | 3.2 | 1.8 | 1.8 | 1.9 | 1.5 | 2.4 | 1.8 |
| 68 | MAPK1 | -1.8 | -1.7 | -2.3 | -2.5 | -1.8 | -1.7 | -1.9 | -2 |
| 69 | MAPRE3 | 3 | 2.7 | 5.3 | 6.5 | 2.6 | 2.7 | 4.1 | 6.1 |
| 70 | MCM3 | -1.8 | -2.8 | -1.5 | -2.2 | -1.7 | -2.2 | -1.9 | -2.9 |
| 71 | MDFIC | -1.8 | -2.5 | -2.4 | -3.1 | -2.7 | -3.2 | -2.3 | -2.7 |
| 72 | MDK | 3.4 | 2.3 | 3.8 | 6.2 | 2 | 1.7 | 2.4 | 2.1 |
| 73 | MICB | 1.9 | 2 | 1.6 | 1.9 | 3.4 | 3.6 | 2.1 | 1.6 |
| 74 | MRAS | -2.5 | -2.7 | -1.7 | -2.2 | -2.4 | -2 | -2 | -2.6 |
| 75 | MRPL3 | -1.6 | -2.3 | -1.7 | -2.7 | -2 | -2.1 | -1.9 | -1.7 |
| 76 | MTHFD1 | -1.6 | -2.8 | -1.9 | -3.4 | -1.9 | -2 | -2.3 | -3.1 |
| 77 | NDRG4 | 1.5 | 2.9 | 2.6 | 5.7 | 2 | 1.8 | 2.1 | 2.1 |
| 78 | NID1 | 1.7 | 2.3 | 2.2 | 5.2 | 1.6 | 2.1 | 2 | 1.7 |
| 79 | NIM1 | 3.8 | 11.5 | 3.6 | 13.1 | 3.9 | 3.6 | 2.3 | 1.8 |
| 80 | NOP56 | -1.7 | -2.5 | -1.8 | -2.9 | -1.8 | -2 | -2.3 | -2.5 |
| 81 | NSRP1 | -1.5 | -1.5 | -3 | -4.4 | -2.1 | -1.6 | -2.4 | -2.2 |
| 82 | NUCKS1 | -1.8 | -2.2 | -1.9 | -2.8 | -2.4 | -2.7 | -1.7 | -2.6 |
| 83 | NUSAP1 | -3.1 | -3.5 | -9.1 | -10.5 | -6.2 | -10.3 | -13.5 | -22.6 |
| 84 | ORAI2 | 3.1 | 2.8 | 3.1 | 3.3 | 2.4 | 1.5 | 1.9 | 1.8 |
| 85 | PDCL3 | -1.7 | -2.1 | -1.5 | -2.5 | -3 | -2.9 | -2.3 | -1.7 |
| 86 | PDS5A | -3.8 | -3.8 | -2.1 | -2.3 | -2.7 | -2.8 | -1.5 | -1.6 |
| 87 | PIK3R2 | 2 | 1.8 | 1.9 | 1.6 | 3 | 2.9 | 2.5 | 2.2 |
| 88 | PKP2 | -2.2 | -2.3 | -2 | -1.7 | -3.2 | -2.5 | -1.5 | -2.7 |
| 89 | PLAGL1 | 2.4 | 2.5 | 1.8 | 2.4 | 5.1 | 3.8 | 2 | 2.2 |
| 90 | PNN | -1.8 | -2.1 | -2.2 | -2.9 | -1.9 | -2.4 | -2.2 | -2.5 |
| 91 | POLD3 | -1.7 | -2.2 | -1.5 | -2 | -2.4 | -2.4 | -2.2 | -2.9 |
| 92 | PPIA | -5.9 | -5.3 | -1.5 | -2.2 | -5 | -7.9 | -2 | -2.3 |
| 93 | PPIP5K2 | -1.5 | -1.9 | -2 | -3.3 | -1.7 | -1.8 | -1.6 | -1.5 |
| 94 | PPM1K | 1.7 | 3.6 | 2.2 | 7.2 | 2.7 | 10.2 | 2.5 | 4.1 |
| 95 | PRC1 | -1.8 | -2.8 | -1.8 | -3.5 | -5.7 | -8.1 | -3 | -5.7 |
| 96 | PTPRU | 2.6 | 1.9 | 2.8 | 3.8 | 2.2 | 1.6 | 2.2 | 4.4 |
| 97 | PVRL1 | 3 | 1.8 | 3.2 | 2.5 | 3.4 | 2.9 | 3.7 | 3.9 |
| 98 | RALBP1 | -10.2 | -11.8 | -12.1 | -14.5 | -7.8 | -12.2 | -5.3 | -6.6 |
| 99 | RALGPS2 | -2.8 | -2.4 | -3.3 | -3.7 | -2.8 | -2.7 | -2.1 | -2.3 |
| 100 | RBMX | -1.5 | -2.2 | -3.5 | -6.1 | -1.6 | -2 | -2 | -3.2 |
| 101 | REEP5 | -1.5 | -1.6 | -1.7 | -2.2 | -2 | -1.9 | -1.6 | -1.5 |
| 102 | RFC5 | -1.5 | -2.1 | -1.8 | -2.9 | -2.3 | -2.5 | -3 | -3.2 |
| 103 | RGL1 | 1.9 | 2.8 | 2.7 | 4.9 | 3.1 | 3 | 2.8 | 3.1 |
| 104 | RNF24 | 2 | 2.5 | 1.5 | 1.6 | 2.2 | 2.9 | 2.9 | 3 |
| 105 | SAE1 | -1.6 | -2.4 | -1.5 | -2.1 | -1.9 | -2.6 | -1.8 | -2.5 |
| 106 | SAMHD1 | 1.6 | 1.5 | 2.4 | 2.9 | 3.9 | 12.5 | 2.4 | 3.5 |
| 107 | SECTM1 | 2.8 | 3.1 | 3.5 | 10.8 | 2 | 2.8 | 2.4 | 3 |
| 108 | SEL1L3 | 1.6 | 2.3 | 1.9 | 4.6 | 1.8 | 1.8 | 1.7 | 2.1 |
| 109 | SEMA7A | 5.2 | 2.9 | 2.5 | 2.5 | 4.2 | 1.7 | 3.4 | 6.9 |
| 110 | SFXN3 | 3 | 3.7 | 2.1 | 2.5 | 2.6 | 3.2 | 2.6 | 2.9 |
| 111 | SHB | 2.5 | 1.6 | 1.8 | 2.3 | 2 | 1.5 | 1.7 | 2.1 |
| 112 | SLC16A2 | 1.7 | 2 | 3.5 | 6.7 | 3.5 | 3 | 5.4 | 6.1 |
| 113 | SLC16A3 | 4.4 | 2.4 | 3.9 | 3.5 | 3 | 1.9 | 1.9 | 2.4 |
| 114 | SLC25A15 | -1.5 | -2.3 | -3.3 | -6.3 | -1.8 | -1.6 | -4.3 | -4.3 |
| 115 | SLC36A1 | 1.8 | 2 | 2.9 | 2.5 | 5.3 | 6.4 | 5.9 | 7.6 |
| 116 | SMARCC1 | -1.8 | -2.3 | -2.5 | -3.4 | -2.1 | -2.7 | -1.6 | -2.5 |
| 117 | SNAP23 | -11.3 | -7.8 | -5 | -6.5 | -5.2 | -4.2 | -2 | -2.3 |
| 118 | SPC25 | -2.4 | -4.3 | -1.6 | -2.6 | -4.1 | -5.8 | -2.9 | -6.7 |
| 119 | SSB | -4.8 | -5.3 | -2.1 | -2.7 | -4.3 | -5.4 | -1.7 | -2.1 |
| 120 | ST13 | -1.9 | -1.8 | -2.1 | -2.3 | -1.7 | -1.8 | -1.5 | -1.5 |
| 121 | STC1 | 5.4 | 9.9 | 2 | 6.3 | 2.7 | 1.7 | 6.2 | 3.3 |
| 122 | STK40 | 3.8 | 2.1 | 2.5 | 2.8 | 2.3 | 2.1 | 1.9 | 2.7 |
| 123 | SYAP1 | -2.2 | -2.3 | -1.8 | -2 | -3 | -3.2 | -1.8 | -2.1 |
| 124 | TBC1D4 | -1.8 | -2.4 | -2.2 | -3.6 | -1.7 | -2.1 | -1.5 | -1.9 |
| 125 | TCEAL4 | -1.6 | -1.6 | -4.6 | -6.4 | -1.6 | -1.8 | -2.7 | -3.1 |
| 126 | TGFBR2 | -2.8 | -2.4 | -2.5 | -1.7 | -3.6 | -3.1 | -2.3 | -2.5 |
| 127 | THOC7 | -1.8 | -2.5 | -1.9 | -2.7 | -3.3 | -3.4 | -2.1 | -2.4 |
| 128 | TIMM10B | -2.3 | -2.2 | -2.1 | -2.6 | -2 | -1.6 | -1.9 | -1.6 |
| 129 | TLR6 | 1.8 | 3 | 1.9 | 1.9 | 4.3 | 9.6 | 6.3 | 8.8 |
| 130 | TMEM194A | -2 | -2.5 | -2.3 | -2.4 | -3.5 | -5 | -2.8 | -3.9 |
| 131 | TMEM230 | 1.6 | 1.7 | 1.5 | 1.8 | 1.8 | 3 | 1.5 | 1.8 |
| 132 | TNKS1BP1 | 2.1 | 1.6 | 2.2 | 3 | 3.2 | 2.9 | 2.6 | 3.7 |
| 133 | TPRG1L | 1.6 | 1.7 | 1.7 | 1.8 | 1.6 | 2 | 1.8 | 2.1 |
| 134 | TRAM1 | -4 | -2.5 | -3.1 | -2.5 | -2.2 | -2.2 | -1.7 | -1.9 |
| 135 | TRIM14 | 2 | 1.7 | 2.7 | 2.6 | 4.3 | 11 | 1.9 | 3.5 |
| 136 | TSPAN9 | 2.2 | 1.6 | 3.4 | 3.6 | 2.7 | 3.1 | 3.2 | 4 |
| 137 | ULBP3 | 2.2 | 2.1 | 2.2 | 1.9 | 3 | 4.1 | 2.2 | 2.4 |
| 138 | VTI1A | -1.6 | -1.7 | -2.3 | -3.3 | -1.8 | -1.7 | -2.2 | -2.4 |
| 139 | WDHD1 | -1.6 | -2 | -2 | -2.7 | -1.6 | -1.6 | -2.7 | -4.4 |
| 140 | ZFP36L1 | 1.6 | 1.8 | 1.5 | 3.3 | 1.7 | 1.8 | 1.8 | 2 |
| 141 | ZFP91 | -1.5 | -1.5 | -2.9 | -3.8 | -1.6 | -1.5 | -2.8 | -3.1 |
| 142 | ZNF106 | -1.9 | -2.3 | -1.9 | -2.6 | -1.8 | -1.8 | -1.8 | -2 |
| 143 | ZNF544 | -1.5 | -1.8 | -2.1 | -2.7 | -4.7 | -3.2 | -2.8 | -2.2 |
| 144 | ZNF664 | -2.2 | -2.7 | -2.2 | -3 | -3 | -2.4 | -1.8 | -2.1 |
